# On the difficulty of validating molecular generative models realistically: a case study on public and proprietary data

**DOI:** 10.1186/s13321-023-00781-1

**Published:** 2023-11-21

**Authors:** Koichi Handa, Morgan C. Thomas, Michiharu Kageyama, Takeshi Iijima, Andreas Bender

**Affiliations:** 1 Centre for Molecular Informatics, Department of Chemistry, University of Cambridge, Lensfield Road, Cambridge, CB2 1EW UK; 2 Toxicology & DMPK Research Department, Teijin Institute for Bio-Medical Research, Teijin Pharma Limited, 4-3-2 Asahigaoka, Hino-Shi, Tokyo 191-8512 Japan

## Abstract

While a multitude of deep generative models have recently emerged there exists no best practice for their *practically relevant* validation. On the one hand, novel de novo-generated molecules cannot be refuted by retrospective validation (so that this type of validation is biased); but on the other hand prospective validation is expensive and then often biased by the human selection process. In this case study, we frame retrospective validation as the ability to mimic human drug design, by answering the following question: *Can a generative model trained on early-stage project compounds generate middle/late-stage compounds *de novo? To this end, we used experimental data that contains the elapsed time of a synthetic expansion following hit identification from five public (where the time series was pre-processed to better reflect realistic synthetic expansions) and six in-house project datasets, and used REINVENT as a widely adopted RNN-based generative model. After splitting the dataset and training REINVENT on early-stage compounds, we found that rediscovery of middle/late-stage compounds was much higher in public projects (at 1.60%, 0.64%, and 0.21% of the top 100, 500, and 5000 scored generated compounds) than in in-house projects (where the values were 0.00%, 0.03%, and 0.04%, respectively). Similarly, average single nearest neighbour similarity between early- and middle/late-stage compounds in public projects was higher between active compounds than inactive compounds; however, for in-house projects the converse was true, which makes rediscovery (if so desired) more difficult. We hence show that the generative model recovers very few middle/late-stage compounds from real-world drug discovery projects, highlighting the fundamental difference between purely algorithmic design and drug discovery as a real-world process. Evaluating *de* novo compound design approaches appears, based on the current study, difficult or even impossible to do retrospectively.

**Scientific Contribution** This contribution hence illustrates aspects of evaluating the performance of generative models in a real-world setting which have not been extensively described previously and which hopefully contribute to their further future development.

**Supplementary Information:**

The online version contains supplementary material available at 10.1186/s13321-023-00781-1.

## Introduction

De novo generative drug design is a current technique of interest [1, 2], not least due to cost pressures [3] and current endeavours to integrate computational and experimental work into Design-Make-Test-Analyze (DMTA) cycles [4]. Looking back, de novo design algorithms have been developed since at least the 1980s [5]. For some time, the mainstream method was the combination of fragment-like building blocks with genetic algorithms [6, 7]. Nowadays, due to the rapid growth of computer hardware including GPU computing, machine learning and deep neural networks applied to molecular generative models have become tractable [8–10].

As with any method, validation–and how to perform validation in a practically relevant manner–has been discussed actively [11]. In the early stages of deep generative models, many researchers only concentrated on how the model produced novel compounds efficiently by copying the distribution of the training dataset, referred to as distribution-learning. Therefore, the principle performance metrics developed were validity, uniqueness, novelty, and diversity which are included in benchmarks such as MOSES and Fréchet ChemNet Distance [12, 13]. However, in a practical drug discovery process, goal-directed optimization is much more important. The gold standard of measuring model performance would be to synthesize and test de novo molecules experimentally (and compare to a baseline control [14]); however, this is intractable for all models considering the experimental resource requirement, given the number of models available and the number of de novo molecules proposed^2^. Recently the CACHE initiative started, whose aim is to validate computationally suggested or generated compounds by experimental testing; however, this activity is limited in scope due to the cost of synthesizing novel structures [15]. In order to fill the need for goal-directed benchmarking, Guacamol [16] has been developed, which contains benchmarks such as rediscovery of and similarity to known active compounds. Although this benchmark is very practical for generative model utilized in lead optimization stage, the dataset is retrieved from ChEMBL [17] and just removes the target compound from the training dataset, where then the task is to rediscover those removed compounds computationally. However, analogues may still remain in the training dataset (of which there are often many, given ChEMBL is constructed from publications which often contain SAR of related compounds), and suggested novel molecules may well be active although *not* being contained in the dataset, and hence also this type of validation has its shortcomings.

A real-world interpretation of generative models in the drug discovery context remains difficult, and the current work attempts to better understand this by retrospectively applying performance measures to generative models applied to public and private drug discovery data sources. The objective of the task is hence to achieve late-stage project compounds, given information from early-stage compounds, in a limited number of steps, and hence in a sample-efficient way (for a more detailed recent evaluation of the sample efficiency of different methods see a recent study [18]). This early/late data split strategy is in analogy to ‘time-split’ validation in the QSAR area, where splitting data into training and test sets along the time domain has been proposed before [19].

**Fig. 1:**
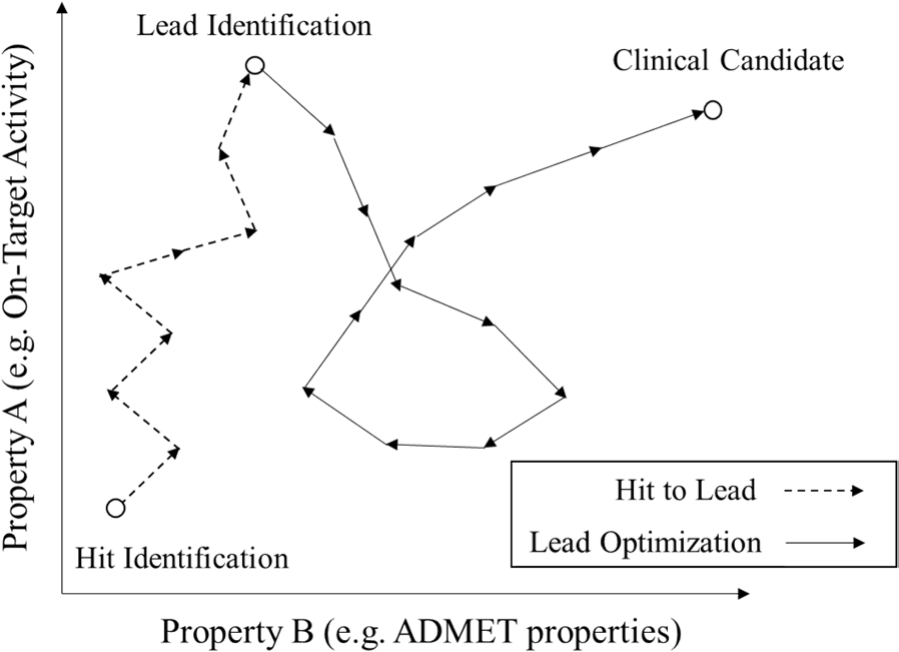
An example of a trajectory of compounds from hit identification to clinical candidate. The white circles and lines (dotted one: hit to lead, and solid one: lead optimization) represent the compound and the trajectory of compound optimization. The X- and Y- axis represent the value of parameters which are better if the values are larger. It can be seen that multiple properties matter in optimization (where in particular the X-axis subsumes a large number of additional properties), and that optimization is usually not linear in practice

However, drug discovery is not ligand discovery, and drug discovery does not only consist of optimizing a single objective in a proxy assay system [20]. More specifically, during the lead optimization stage of a drug discovery project, multiple-parameter optimization (MPO), for parameters such as primary target activity, activity against off-targets, and also physicochemical and ADME properties such as permeability, intrinsic clearance, solubility etc. need to be optimized simultaneously [21], an area which has found consideration only in few computational studies [22, 23]. In reality the MPO process is very complicated in a drug discovery project, because the target profile could be easily changed (and even multiple times) during the course of a project, where new problems appear every step along project progress (Fig. 1) [24]. In this work, we attempted to see whether generative models can be validated retrospectively, on the one hand with public data mapped onto a pseudo-time axis, and on the other hand with real-world project data from different projects in a pharmaceutical company.

Regarding the architecture of the deep generative model, we decided to use one of the widely used approaches in the field, namely REINVENT [25, 26]. Recently, many architectures of generative models for de novo design have been published such as recurrent neural network (RNNs) [27], convolutional neural network (CNNs) [28, 29] and graph convolutional neural network (GCNN) [30]. In the drug discovery field, although there are many models including variational auto encoder (VAE) [1], and Generative Adversarial Networks (GAN) [31], due to the success of NLP, which was driven by many techniques like long-short time memory (LSTM) [32], gated recurrent unit (GRU) [33] and attention mechanism [34], language models have found great resonance [22, 23, 35, 36]. We chose REINVENT as the generative model in this study, an RNN-type language model with the ability to perform goal-directed optimization through fine-tuning and reinforcement learning, due to its availability, flexibility and wide use making results herein more relatable [37–43].

## Materials and methods

### Dataset

**Fig. 2:**
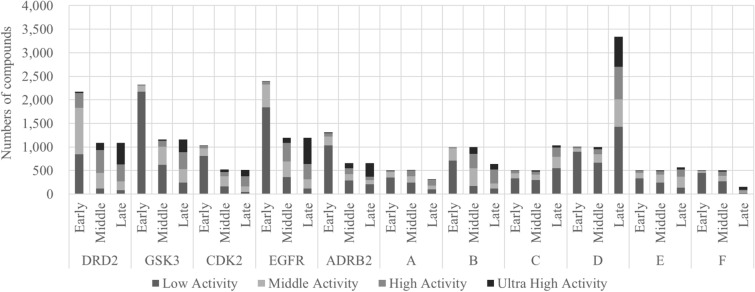
The datasets used in this study include wide range of activity. The thresholds for activity classes generally are pXC50 values of less than 6 for low, over 6 to less than 7 for middle, over 7 to less than 8 for high, and over 8 for ultra-high compound activity

For the public dataset, a variety of targets with differing mechanisms of action were chosen such as G protein-coupled receptors (GPCR), kinase, and kinase receptor; data for five targets were selected from Excape-DB [44], namely for DRD2 (Dopamine Receptor D2, 4341 active compounds), GSK3 (Glycogen synthase kinase 3, 4646 active compounds), CDK2 (Cyclin-dependent kinase 2, 2065 active compounds), EGFR (Epidermal Growth Factor Receptor, 4777 active compounds), and ADRB2 (Adrenergic receptor β2, 2616 active compounds). The quantitative rationale was to select datasets which have been well studied in previous publications and which include more than 1000 compounds individually with pXC50 values (Additional files 1, 2, 3, 4, 5: Dataset 1 to 5). For the in-house dataset, six projects were collected from TEIJIN Pharma’s in-house database which also include more than 1000 compounds individually. These are named here as A, B, C, D, E, and F. Figure 2 and Additional file 6: Table S1 show the number of compounds for each dataset, separated by activity values and ‘early’, ‘middle’ and ‘late’ stage annotations, further details of which are explained in the following.

### Time series pre-processing

#### Public dataset

The public dataset utilized in this study was derived from ExCAPE-DB. All targets are well-studied and include more than 1000 bioactivity data points with pXC50 values. The simplified molecular-input line-entry system (SMILES) strings were obtained from ExCAPE-DB for all molecules, and canonicalized using the RDKit (version 2020.09.01) component “RDKit Canon SMILES” and “Speedy SMILES De-salt” in the KNIME (version 4.3.4). However, this public database contains no ‘project registration date’ and the compounds deposited in the underlying databases (ChEMBL [17] and PubChem [45]) are usually done by publication or grouped upload, not reflecting realistic project time series optimization. Therefore, in order to mimic the time series of a practical drug discovery process that increases the activity with time elapsed, data was mapped onto a ‘pseudo-time axis’ as follows. We transformed the data by principle component analysis (PCA) using Datawarrior (ver 5.2.1) [46], and then the following three steps. (1) The canonical SMILES of the public dataset were input to calculate the FragFp [47] fingerprints. The FragFP fingerprints were used to calculate the normalized PCA components of 3 components. (2) Then, these components alongside pXC50 value of each compound (in total 4 dimensions) was used to obtain another PCA score of 3 components. These 3 final PCA components hence include information on both similarity of compounds in fingerprint space as well as bioactivity. (3) Finally, the Euclidean distance of all compounds in each dataset to the compound that has lowest pXC50 value was calculated using the final 3 PCA components. This process introduces an ordering of compounds in bioactivity space (from low to high potency), as well as chemical space (from a low potency starting point, to high potency compounds with increasing dissimilarity to the starting point). We are aware that this process does not necessarily resemble a real-world drug discovery project, but it at least represents compound progression towards higher-potency compounds, which, given the limited availability of public domain timestamped project data is the only practically feasible option we were able to identify for a public dataset. Then, the datasets were divided by both the activities and pseudo-stages.

**Fig. 3:**
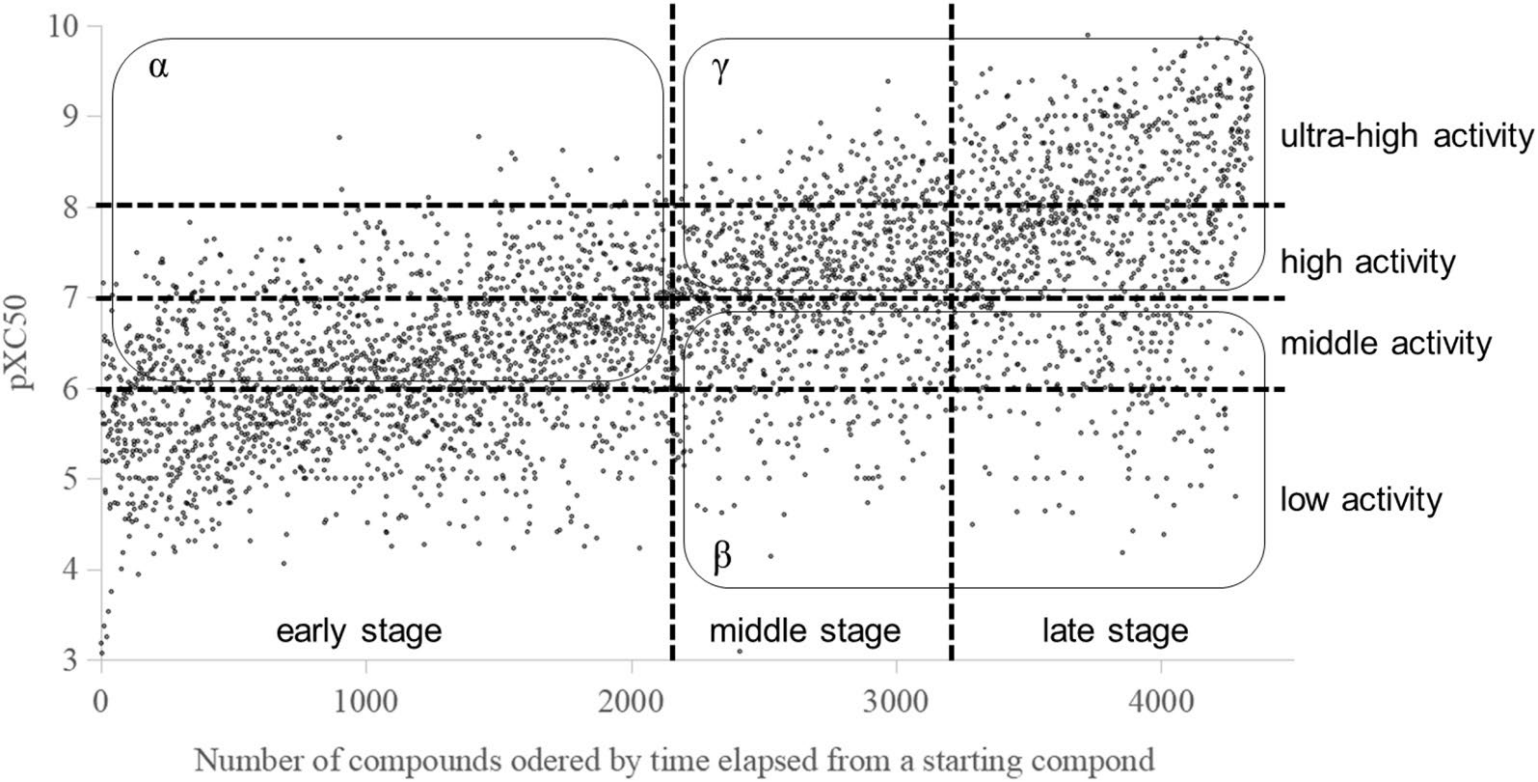
An example of data division according to stages and bioactivities. The region of α that consists of more than middle activity compounds in the stage of early corresponds to the training dataset for fine-tunning to produce focused agent. The region of β consists of low and middle activity compounds in the middle and late stage, and the region of γ consists of more than high activity compounds in the middle and late stage. The X-axis is unitless

To categorize the activities, pXC50 thresholds for activity classes in most projects are less than 6 for “low”, over 6 to less than 7 for “middle”, over 7 to less than 8 for “high”, over 8 for “ultra-high”. To categorize the stages for public projects already transformed into a pseudo time-series, those from the beginning of the compounds to 50%, 25% (accumulated from 50 to 75%), and 25% (accumulated from 75 to 100%) were classified into early, middle and late stage, respectively. As an example, Fig. 3 shows the DRD2 dataset, with compounds classified across the different stages of the drug discovery ‘project’ mapped onto the pseudo-time axis, as well as the different bioactivity ranges used in this work.

#### In-house dataset

The in-house dataset was retrieved from TEIJIN Pharma Ltd’s Database, and we selected 6 projects (A to F) which have more than 1000 bioactivity datapoints of pXC50 values. The date of completed synthesis for in-house compounds was recorded in the TEIJIN database for a given project. Consequently, being different from public dataset, we directly used the date for the time-series of the in-house dataset. An additional difference from the public dataset was that we know there was at least one additional property to be improved for each project that was not on-target activity, such as metabolism, physicochemical properties, etc., and this can even change at different timelines of the project [21]. Hence a second objective of using this dataset, apart from benchmarking generative models, was to evaluate to what extent the structures generated by de novo generative models actually follow the optimization trajectory across a set of real-world drug discovery projects. Regarding data classification along the bioactivity axis, for projects A, B, C, D, the setting of activity classification was the same as public dataset. However, for projects of E and F, given the bioactivity distribution in those cases, thresholds for activity classes have been set to less than 7 for “low”, over 7 to less than 8 for “middle”, over 8 to less than 9 for “high”, over 9 for “ultra-high”. As for the classification of stages of in-house projects, 500 or 1000 was selected based on the progression of bioactivity in order to evenly split activity groups.

### Regional classification and similarity analysis

In order to establish ‘project progress’ with respect to potency, we next defined intervals α, β and γ (as shown in Fig. 3 for the DRD2 dataset) in bioactivity space as follows. The region α is compounds with over middle activity in the early stage, while the region β is compounds with low and middle activity in the middle and late stage and region γ is compounds with high and ultra-high activity in the middle and late stage. We then analyzed progression in the bioactivity domain across different project stages (*i.e.*, to which extent potency could be optimized by the model) by calculating the average similarity of generated molecules to the single nearest neighbour (aSNN) present in a given part of the dataset, based on the Tanimoto similarity of Morgan fingerprints using RDKit [12, 48] and with the aSNN calculations performed as implemented in MolScore [39].

### Model training

**Fig. 4:**
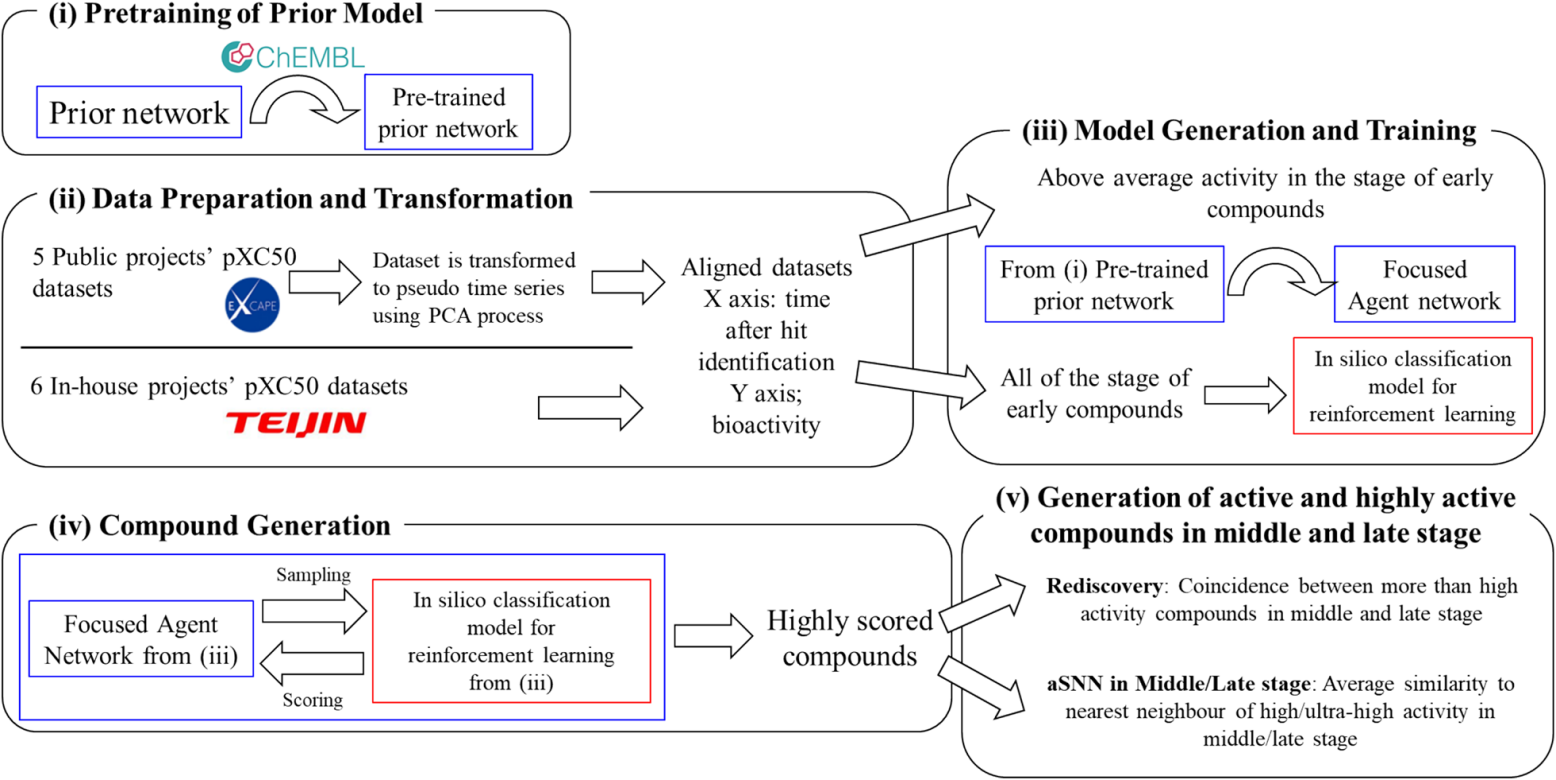
Workflow of this study (for details see main text). As options, Inception and diversity filter (DF) could be used in the sampling process of (iv).

We used REINVENT 2.0 (from https://github.com/MolecularAI/Reinvent accessed 14/09/20, now corresponding to branch reinvent.v.2.0) [26] as a de novo generative design strategy, given its wide use in the field [49]. Figure 4 shows the workflow of this study using the REINVENT framework, which is described in more detail as follows:

#### (i) Pretraining of prior model

Compounds were prepared in accordance with the REINVENT pipeline [26] as standardized non-isomeric SMILES. The Prior network was pre-trained on a dataset of 1,442,368 compounds derived from ChEMBL where the molecules were restrained to containing between 10 and 50 heavy atoms and elements {H, B, C, N, O, F, Si, P, S, Cl, Br, I} [17]. Only for public dataset, the compounds included in the ChEMBL dataset were omitted. In the pre-training, the Prior network was trained for a total of 10 epochs with a batch size of 128 with an adaptive learning rate starting from 0.0005. All other settings were set to default [26]. All neural network training was conducted on an NVIDIA GeForce GTX 1650 Ti.

#### (ii) Data preparation and transformation

We next utilized the dataset of each project (either public or in-house) for fine-tuning, whose compounds were also processed as described in (i). Since the purpose of fine-tuning is to focus on the higher activity compounds, the compounds chosen for this step were early-stage compounds with above-average activity (region α).

#### (iii) Model generation and training

##### For focused agent network

The Pre-trained prior network obtained in (i) was fine-tuned by the compounds prepared in (ii), and the model obtained here was called the Focused Agent network. During fine-tuning, the pre-trained prior network was trained for a total of 10 epochs with a batch size of 128 with an adaptive learning rate starting from 0.0005. The other settings were adopted as default [26].

##### Random forest model for the reinforcement learning scoring function

All compounds in the early stage were used to build a classification model which was used as the scoring function for reinforcement learning (RL) to optimize. Compounds possessing above average activity were classified as active and those below average activity classified as inactive. The dataset was divided into 70% training and 30% test, and ECFP6 descriptors [50] (1024 bit, radius: 3) were generated using RDKit (version 2020.09.01) Chem functions [48] while a Random Forest (RF) (Python (ver. 3.7.10), scikit-learn (ver 0.24.2) library RandomForest) ensemble.RandomForestClassifier function was used for machine learning [51] (Additional file 6: Fig. S1). The parameters of RF were set as follows; max_depth: 20, n_estimator: 100, others: default setting. RL was performed for 500 steps with a batch size of 128, a sigma value of 128 and learning rate of 0.0001.

#### (iv) Compound generation

The compounds were generated from the Focused Agent network from (iii) and scored by the in silico classification model from (iii) repeatedly as the RL framework. 5000 de novo molecule were sampled in total from the final network. The highest-ranked 100 and 500 compounds were selected according to the in silico classification score for subsequent analysis.

#### (v) Evaluations

As basic metrics, validity, uniqueness and novelty were calculated for all runs which have also been used in previous work [12, 16]. Validity is the fraction of correctness that a SMILES string translates to a real structure. Low validity is indicative of a poorly behaving model that has struggled to learn the SMILES grammar. Uniqueness is the fraction of unique molecules, where non-unique molecules are defined as having canonical SMILES that match those previously sampled or in the same batch. Low uniqueness is indicative of a poorly behaving model that is ‘stuck’ in a particular region of chemical space. Novelty is the ratio of valid, unique canonical SMILES not present in the training dataset (pre-training: ChEMBL, fine-tuning: above average activity compound in the early stage of each project which locates in region α), and low novelty indicates the model cannot generalize beyond the training data, which is precisely the aim of de novo design. All these calculations were implemented in Python 3.7.10 using the original code following the equations below, Eq. 1–3, where *Ngen* represents the number of generated compounds, *Nval* represents the number of valid compounds, *Nuni* represents the number of unique compounds in generated compounds, and *Nunk* represents the number of unknown compounds in generated compounds [52].

1 $$Validity\;(\%) =\frac{Nval}{Ngen}\times 100$$

2 $$Uniqueness\;\left(\%\right)=\frac{Nuni}{Nval}\times 100$$

3 $$Novelty\; (\%)=\frac{Nunk}{Nuni}\times 100$$

Finally, the highest-scored compounds selected from (iv) were evaluated by the following metrics. The calculation of these metrices was implemented in Python 3.7.10.

1. Rediscovery ratio, defined as Eq. 4, in order to assess whether experimentally confirmed highly- or ultra-highly active compounds were generated, where *Nredis* represents the number of generated compound which agreed with the real high or ultra-high activity compound in the middle or late stage.
  4 $$Rediscovery\; \left(\%\right)=\frac{Nredis}{Ngen}\times 100$$
2. aSNN in the middle stage, to evaluate whether generated compounds were similar to compounds from the middle stage of a given project with high or ultra-high activity.
3. aSNN in the late stage, to evaluate whether generated compounds were similar to compounds from the late stage of a given project with high or ultra-high activity (which means that the generative model behaves similarly to ‘real world’ projects, to the extent captured by the data used in this work and at that stage).

### Generative models and options

#### Control experiment: prior network only

As a baseline to compare to RL we generated compounds from the pre-trained prior network only, called “Control” in the following. The aim of this baseline was to investigate the effect of fine-tuning and reinforcement learning using the dataset prepared in this study.

#### Focused learned agent network (FL)

We generated compounds from focused learned agent network, namely these compounds were generated without the effect of reinforcement learning.

#### Reinforcement learning (RL)

This represents the ‘vanilla’ approach of this work, employing only Reinforcement Learning.

#### Diversity filters (DF)

The next variation was the use of a “diversity filter (DF)” [26], which has been shown before to give an increase in the structural diversity of compounds generated [42, 53]. The parameters of DF were set to default as follows; name: IdenticalMurckoScaffold, nbmax: 25, minscore: 0.4. This run is called “RL-DF”.

#### Inception

The purpose of “Inception” [26] is to keep track of previously well scored compounds and to randomly expose a subset of them to the agent, thus helping to direct the learning. The parameters of Inception were set as follows; memory_size: 20, sample_size: 5. In this study, 30 compounds that were at least of’high’ activity in early stage were used. (It is noted that although we used inception mode in any runs, since we set sample_size: 0 other than “Inception” run, inceptions were not executed.)

Consequently, there are five different ways the generative model was run, which were Pre-trained prior network (Control), RL, RL-DF, RL-Inception, RL-DF-Inception.

### Compound clustering

To investigate the profiles of the activity of real (public and in-house) compounds according to the time elapsed quantitatively, we used compound k-means clustering as implemented in sklearn.cluster using ECFP6 fingerprints calculated using RDKit [48] and cluster size: 10. Then, we counted the number of compounds in each cluster and in each region (α, β and γ in Fig. 3).

Furthermore, to understand the chemistry of generated compounds, we examined it by visual inspection, using DRD2 compounds as an example. From each cluster the centroid structure of each cluster was selected as a representative, and the structure which has the highest pXC50 value was selected as the highest-scoring structure of its cluster.

### Negative log likelihood

To investigate the real compounds located in α, β and γ are reflected in the agent network, we calculated negative log likelihood (NLL) using the compounds in α, β and γ, separately. The function in REINVENT 2.0 (likelihood_smiles function from models.py)^26^ was used.

## Results and discussion

### Dataset characterization across the bioactivity and time domain

**Fig. 5:**
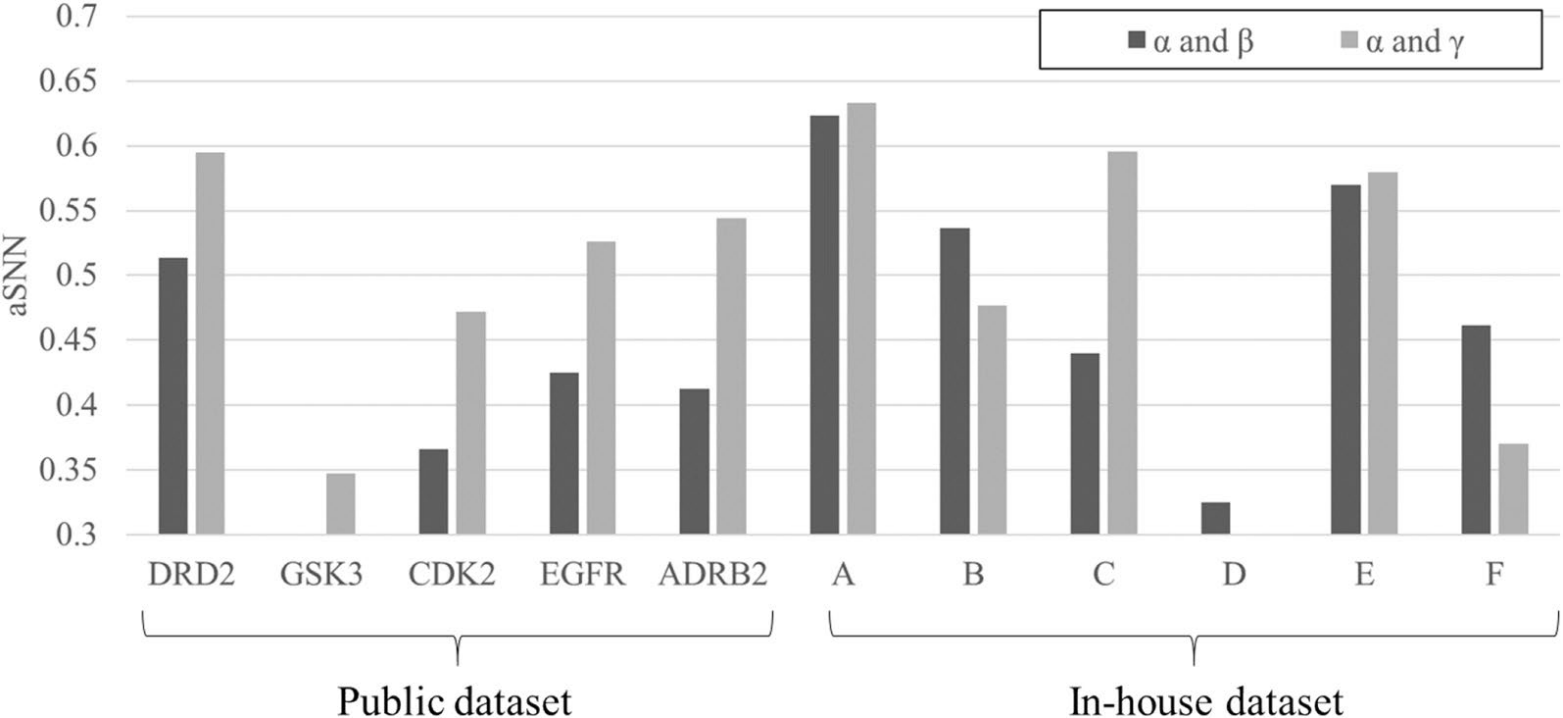
Average of single nearest neighbour similarity (aSNN) between training and test compounds. The aSNN for all projects for low or high activity real compounds were largely different from public and in-house projects. It can be seen that the profiles in Public dataset (aSNN of α-β < α-γ) was different from in-house (mostly, aSNN of α-β > α-γ). The cut-off values of aSNN considered similar was set to be 0.3

We firstly aimed to understand the distribution of our datasets across the time and bioactivity domain, the results of which are shown in Fig. 5. For the public projects, the aSNN between α and γ are much higher (by around 0.1) than those between α and β. However, for the in-house projects, the aSNN between α and γ were mostly similar to, or lower than, values between α and β except for project C. The underlying reason is likely that chemical series from publications including high-activity ligands were quite different from those with lower activities (hence giving area β a different composition), which is the result of different ligands (which different activity) being reported in different publications, w.r.t. both chemistry and publication date, given that those were the criteria used for dataset assembly here. On the other hand, for the in-house dataset this wasn’t really the case, meaning that in relatively more cases late-stage high-activity space was still in a chemical area similar to that occupied at project start (although the situation is quite different for different projects). It can clearly be seen that both classes of datasets hence behave differently, which is entirely expected from the way they were constructed (see methods section for details).

### Metrics of generated compounds (validity, uniqueness, and novelty)

Next, we calculated the validity, uniqueness, and novelty of the generated compounds. The results for RL are shown in Additional file 6: Table S2. The validity for each target was over 98%. The uniqueness of generated compounds for the public dataset was relatively high, from 39.4% (GSK3) to 82.2% (DRD2), while the corresponding value for the in-house datasets was much lower, ranging from 15.1% (project F) to 50.9% (project E). The novelty of each target was over 70% (for detailed results see Additional file 6: Tables S3–5). Through all the runs, regardless of targets, the validity and novelty were high enough, over 95% and 70%, respectively, which are appropriate in practice. The lower uniqueness values in in-house datasets ranging from 15.1% (project F) to 50.9% (project E) might reflect more congeneric compounds used in focused learning compared to the combination of different publications in public datasets. Across projects, the uniqueness of the RL-inception runs were lower than the other runs, from 36.4% (GSK3) to 59.8% (DRD2) for the public dataset, and from 19.4% (project F) to 40.8% (project E) for the in-house dataset which is lower than for the original RL runs. However, if the DF was used as an option, the low uniqueness was completely recovered, both for the RL-DF run, as well as the combination with Inception, with values ranging from 99.0% (project B) to 99.8% (CDK2) for the RL runs, while values for RL-DF-inception ranged from 96.8% (project B) to 98.5% (DRD2 and ADRB2). This underlines the importance of using diversity filters to ensure uniqueness of generated structures across the different situations considered here [40].

### Rediscovery

**Fig. 6:**
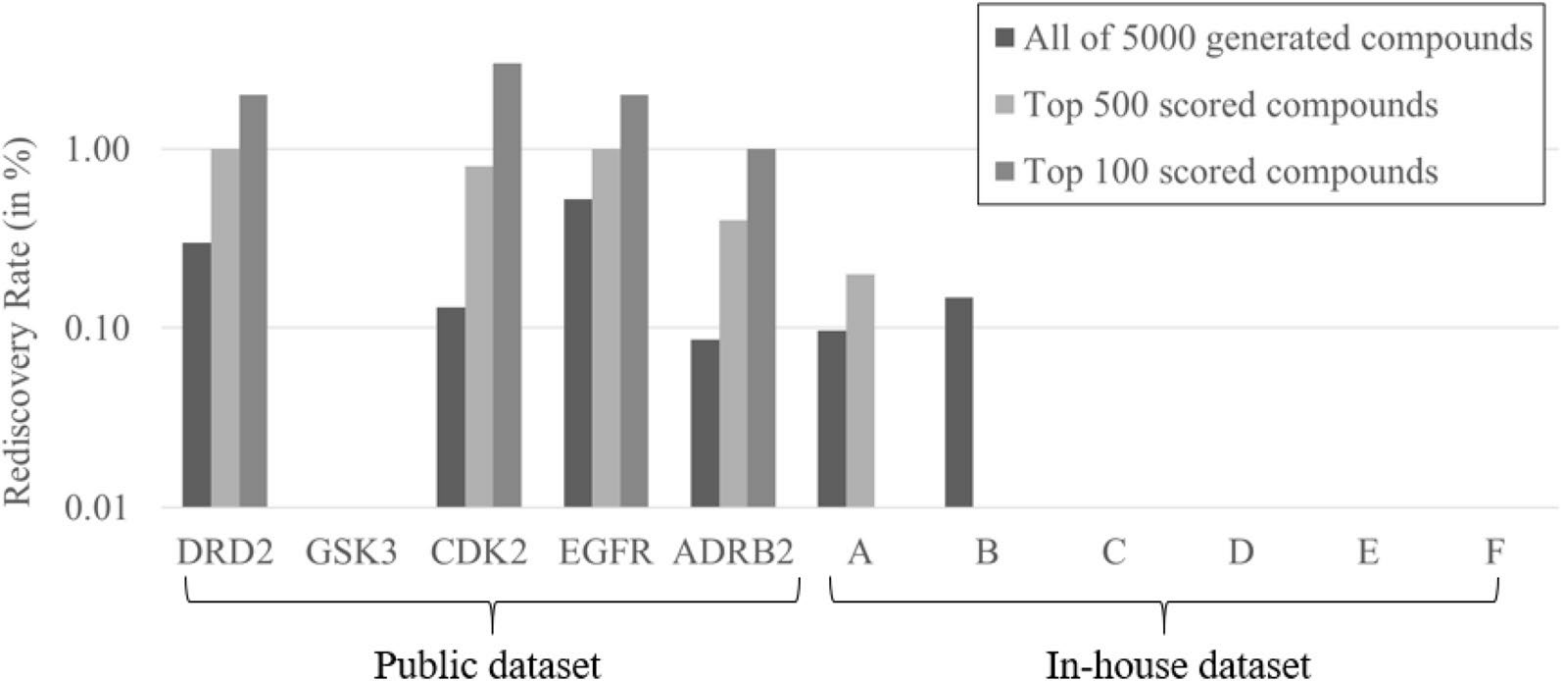
Rediscovery of compounds was higher for public projects than in-house in the reinforcement learning (RL) setting. For further details see Additional file 6: Table S6

We next analyzed the rediscovery rates of generated compounds using RL alone, the results of which are shown in Fig. 6. For public projects, other than GSK3, we could find compounds identical to real high activity compounds. The percentage of rediscovery for DRD2, CDK2, EGFR, and ADRB2 were 0.30%, 0.13%, 0.52%, and 0.09% for all 5,000 generated compounds, respectively; when using the in silico classification score for ranking 1.0%, 0.8%, 1.0%, and 0.4% of the 500 highest-scored generated compounds represented known actives, while this was the case for 2%, 3%, 2%, and 1% for the top 100 scored generated compounds, respectively. For in-house projects, only the generative models for project A and B could find identical compounds to the real high activity compounds. The percentage of rediscovery in project A and B were 0.10%, and 0.15% for all 5,000 generated compounds; when using in silico classification scores for further selection 0.20% and 0.00% of the top 500 scored generated compounds represent known actives, while the top 100 scored generated compounds had no rediscovery (more details are shown in Additional file 6: Table S6). This decrease of rediscovery and lack of enrichment achieved by additional ranking of in silico classification model indicates that prospective performance may be poor, and indeed in the case of project B retrospective performance was marginally better than random (Additional file 6: Fig. S1). Hence, we consistently find that rediscovery was much higher for public projects than in-house projects. Rediscovery was less than 1% for all generated compounds, and less than 3% for the top 100 scored compounds (Fig. 6), which is significantly lower than in a previous study: In work by Segler et. al. [54] where the rediscovery ratio was around 10% for two bioactivity endpoints (which were growth inhibition endpoints though, namely inhibitory activity for *Plasmodium falciparum* and *Staphylococcus aureus*). However, methodological differences exist: In this previous study the test dataset was selected randomly and removed from the training dataset, which means that congeneric compounds might still exist in the training dataset, and then the generative models were fine-tuned. This explains the high rediscovery rates; however, this situation doesn’t really resemble a real-world drug discovery situation. On the other hand, in a study performed by Atance et. al. [55], which removed a test dataset for DRD2 completely from the training dataset, the percentage of rediscovery was less than 1%; the condition of this study was more similar to ours with respect to conditions and results obtained.

We found that rediscovery (the percentage of known actives present in the de novo-generated compounds), was greater in public projects (1.60%, 0.64%, and 0.21% of the top 100, 500, and all 5,000 generated compounds, respectively) than that in in-house projects (where the values were 0.00%, 0.03%, and 0.04%, respectively). This shows that the public dataset which was mapped on a pseudo-time axis behaves fundamentally different from a real-world drug discovery project, leading to very different numerical results.

### Similarity Analysis of generated compounds to middle stage compounds

**Fig. 7:**
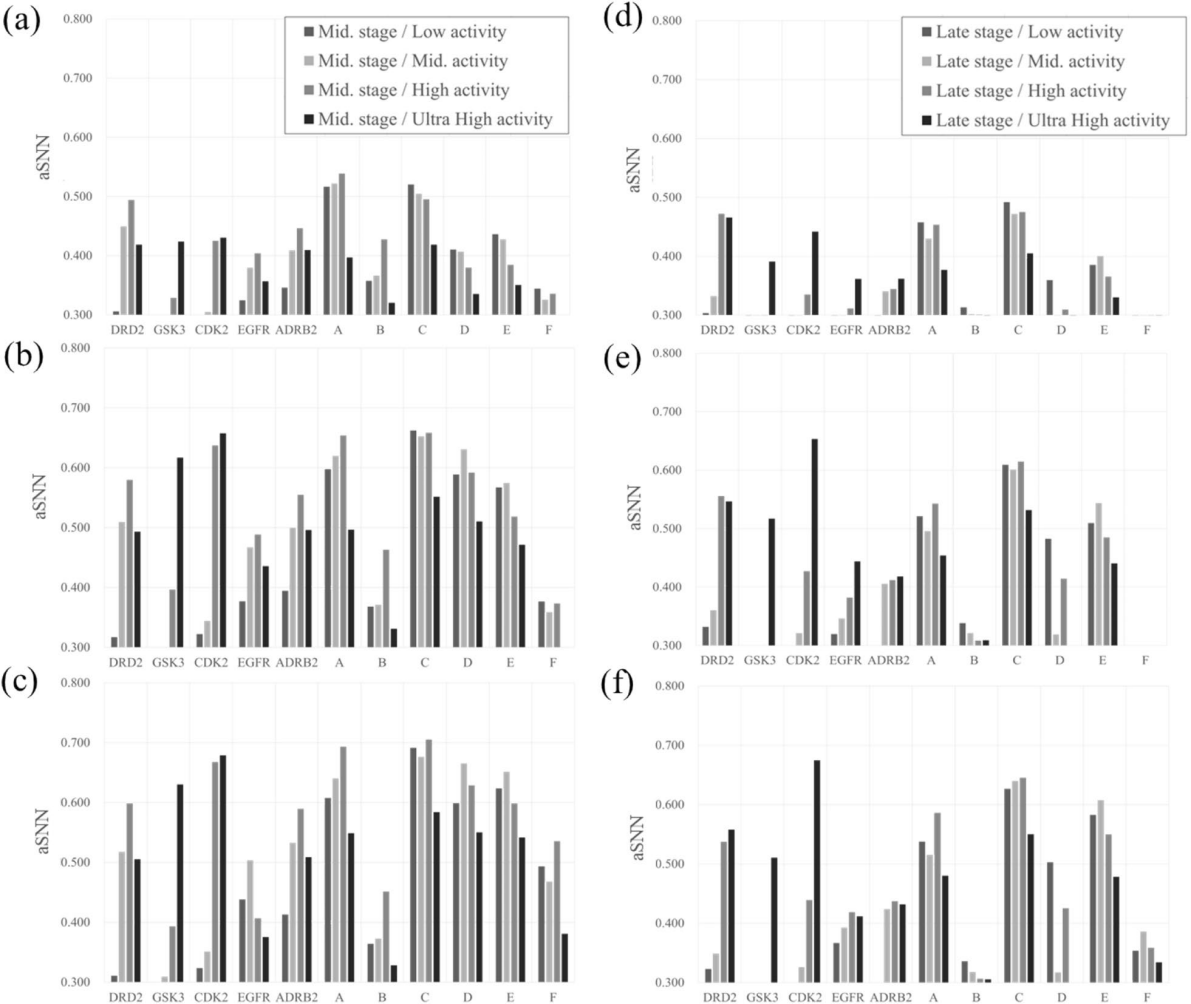
Average of single nearest neighbour similarity (aSNN) between generated and middle/late stage’s test compounds. The aSNN between generated compounds from all projects in reinforcement learning (RL) for (**a**, **d**) all 5,000 compounds generated, for (**b**, **e**) the highest-scored 500 compounds by an in silico classification model, and for the (**c**, **f**) highest-scored 100 scored compounds by an in silico classification model to the real compounds in middle (**a** to **c**) or late (**d** to **f**) stage are shown. From a to c, it can be seen that activity model selection generally increases aSNN, with the magnitude of the effect widely varying across projects, from d to f, generally speaking, values are lower than in a to c (for middle-stage compounds), and hence long-term compound evolution is much more difficult to model than short-term compound evolution. The cut-off values of aSNN considered similar was set to be 0.3

To investigate whether the generative model can produce compounds similar to known actives, we next calculated the aSNN (average Similarity of the Nearest Neighbour) between the generated compounds and known active compounds. The aSNN between generated compounds and the real compounds which belong to the middle stage are shown in Fig. 7a to c (all the value of aSNN can be seen in Additional file 6: Table S7). For the public projects, given all of 5,000 generated compounds, aSNN through the projects of high/ultra-high activity compounds were much higher than that of low/middle activity compounds (the average of aSNN across projects for low/middle/high/ultra-high activity was 0.304/0.367/0.420/0.408, respectively) [56]. Hence, for the public dataset, and given the particular way this dataset was constructed, optimization towards the single objective of primary target activity was possible. For the in-house projects those trends were inconsistent (the average of aSNN across projects for low/middle/high/ultra-high activity was 0.431/0.425/0.427/0.348, respectively). For project A and B the aSNN to high activity compounds was higher than the corresponding value for low/middle activity compounds; however, for projects C to F the aSNN of generated compounds to high/ultra-high compounds was conversely lower than that to low/middle activity compounds (Fig. 7a). We can hence conclude that both datasets behaved very differently: While for the public dataset evolution towards the chemical space of higher-potency compounds was generally possible, this was not the case for the in-house projects analyzed.

Next we analyzed the compounds selected by the in silico classification model to investigate the effect of this data processing step. For the public dataset we found that the aSNN of the 500 compounds highest ranked by the in silico classification model was much higher than when all generated compounds were used, namely the compounds generated were more similar to the ultra-high activity compounds (the average of aSNN through projects for low/middle/high/ultra-high activity was 0.329/0.424/0.531/0.540, respectively). For the in-house projects the compounds generated were more similar to the high activity compounds as well, but this is not the case for ultra-high activity compounds (the average of aSNN through projects for low/middle/high/ultra-high activity was 0.527/0.534/0.543/0.442, respectively). This trend held for the 100 compounds highest ranked by the in silico classification model, where the aSNN across projects for low/middle/high/ultra-high activity was 0.343/0.443/0.531/0.540 (for public data) and 0.563/0.579/0.602/0.489 (for in-house data), respectively. Especially for GSK3 and CDK2, the aSNN of high/ultra-high compounds was more than two times higher than when using all generated compounds (Fig. 7b, c). Furthermore, in project C and F when using top 100 scoring, the aSNN of high activity compounds were higher than that of low/middle activity compounds (Fig. 7c). Hence we can conclude that filtering by an in silico classification model has an overall beneficial effect to select higher activity compounds across most of the public and in-house datasets, with the magnitude of the effect widely varying.

### Similarity analysis of generated compounds to late stage compounds

Next, we analysed the aSNN between generated compounds and the real compounds which belong to the late project stage, which usually means both greater chemical evolution, and more bioactive (or generally optimized, with respect to the objective properties) compounds. Generally speaking, the assumption was that the more time elapsed, the more difficult it will be for the model to generate compounds similar to real late-stage project compounds. The results of this analysis are shown in Fig. 7d to f (all the value of aSNN can be seen in Additional file 6: Table S7). It can be seen that the value of most aSNNs was lower than those in the middle stage (Fig. 7a–c). In the public projects, given all of 5,000 generated compounds, the aSNN of generated compounds to high/ultra-high activity compounds was higher than that to low/middle activity compounds (the average of aSNN values across projects for low/middle/high/ultra-high activity being 0.259/0.297/0.341/0.404, respectively). However, this was not the case with the in-house projects, with the average aSNN values across projects for low/middle/high/ultra-high activity being 0.376/0.357/0.361/0.311, respectively (Fig. 7d). Hence, we can conclude that again both types of projects behave differently; for the public dataset evolution towards the chemical space of higher-potency compounds was generally possible, but not so for the in-house projects.

Then, to investigate the effect of score filtering, we analyzed the top scored of the generated compounds. Although the absolute value of aSNN was higher when we using top 500 (with the average aSNN across projects for low/middle/high/ultra-high activity being 0.281/0.336/0.402/0.516 for the public datasets and 0.455/0.429/0.441/0.370 for the in-house datasets) as well as the top 100 compounds (with the average of aSNN across projects for low/middle/high/ultra-high activity being 0.289/0.349/0.414/0.517 for the public datasets and 0.490/0.464/0.479/0.394 for the in-house datasets), there were not as drastic changes compared to those in the middle stage when performing the same analysis (Fig. 7b, c, e, f). We can conclude that score filtering in the late stage is more difficult compared to the middle stage, and this might be derived from the greater time elapsed (and hence chemical space evolving) since the generation of the models.

In absolute terms, a similarity above ca. 0.3 in ECFP4/Tanimoto space (as a broad rule of thumb) often indicates similar bioactivity [57], which was in many cases reached by the current projects. Hence, in absolute terms, it seems that the chemistry generated should be suitable for drug discovery.

However, public and in-house projects behaved vastly different throughout the current analysis, which is understandable given the differences in how both datasets were constructed. For in-house datasets, the aSNN were mostly higher than 0.3; however, the aSNN to the real high or ultra-high active compounds was across projects consistently lower (Fig. 7) than to the real low or middle active compounds. This could be influenced by the somewhat artificial setup of this study, where we focused on a single objective, namely on-target activity; however, during any practical drug discovery project the consideration of multiple (and often competing) objectives is inevitable [20]. This is supported by the analysis shown in Fig. 5, where it is clear that in real projects compound evolution does not simply follow an optimization of on-target activity. Consequently, it is more difficult to reproduce a compound trajectory from real-world project data, compared to that of just optimizing on-target activity, which is what we consistently also observe from our results in this study; our setup (single property optimization) is a potential limitation to consider during interpretation, especially of in-house projects.

### The effect of in silico classification model accuracy on rediscovery and aSNN

Next, we investigated the relationship between in silico classification model predictivity, learning curves and rediscovery/aSNN, to evaluate whether better bioactivity models also lead to higher values across these performance measures. The results of this analysis are shown in Additional file 6: Fig. S1a, where balanced accuracies were acceptable for use in practice. We also investigated the relationship between the classification accuracy and rediscovery/aSNN (to the late stage compounds with high/ultra-high activity) in Additional file 6: Fig. S2. It can be seen that there is a positive correlation to aSNN (r^2^ values from 0.249 to 0.463), therefore, a more accurate classification model is more likely to result in higher aSNN of de novo compounds than a less accurate one. However, this is not guaranteed due to it being only a weak correlation. Moreover, we investigated the resulting scores of the in silico classification model on the real compounds in sub-groups α, β and γ, shown in Additional file 6: Fig. S3. Unsurprisingly, the α compounds are scored highest as they are also the training compounds representing the positive class. The compound scores for γ were higher than β in all projects except in-house projects D and F, suggesting that these models could guide RL to high activity compounds more frequently than low activity. Finally, we checked the learning curve for each target in Additional file 6: Fig. S4 and found they could reach the higher scores close to 1.0. However, the representative scores for the γ sub-group were objectively lower (0.1–0.8) than RL-achieved scores (usually 0.7–1.0). This highlights that optimizing for high-scoring molecules is not necessarily going to generate molecules from the same distribution as the γ sub-group. In practice, this is not known apriori and exemplifies the difficulty in applying such routine QSAR models that may contain unexpected applicability domains, for example, predicting high-activity (γ sub-group) molecules with relatively low probabilities of activity. It is also worth noting that low predicted probabilities of activity do not necessarily translate to a negative prediction, as the optimal classification threshold may exist anywhere in the 0–1 range [58]. Therefore, maximizing the binary label [0, 1] as a reward in RL based on an optimal decision threshold instead of the predicted probability may result in higher aSNN in these cases.

### The effect of reinforcement learning on rediscovery and aSNN

To investigate the low performance on rediscovery, we evaluated RL thoroughly. Here, we compared the rediscovery and aSNN in FL and RL across all of targets and projects to investigate the effect of RL (Fig. 7, Additional file 6: Table S6, Figs. S5,6). On the one hand, regarding the rediscovery, we could not reach a distinctive conclusion; the values in FL for all of 5,000/top 500/top 100 generated compounds were 0.13/0.80/3.00 (public dataset), 0.01/0.10/0.00 (in-house dataset), and those in RL were 0.21/0.64/1.60 (public dataset), 0.04/0.03/0.00 (in-house dataset) (Additional file 6: Table S6). The absolute values of the rediscovery were too low to compare and lead to a conclusion.

On the other hand, regarding aSNN we could obtain another perspective. The comparison of aSNN results for low/middle/high/utra-high activity compounds of FL and RL in public dataset were as follows; all of 5,000 generated against middle stage compounds: 0.280/0.301/0.301/0.268 (FL) and 0.304/0.367/0.420/0.408 (RL), top 500 generated against middle stage compounds: 0.317/0.402/0.433/0.397 (FL) and 0.329/0.424/0.531/0.540 (RL), top 100 generated against middle stage compounds: 0.332/0.449/0.519/0.476 (FL) and 0.287/0.326/0.403/0.490 (RL); all of 5,000 generated against late stage compounds: 0.243/0.255/0.275/0.287 (FL) and 0.259/0.297/0.341/0.404 (RL), top 500 generated against late stage compounds: 0.274/0.304/0.362/0.407 (FL) and 0.281/0.336/0.402/0.516 (RL), top 100 generated against late stage compounds: 0.343/0.443/0.531/0.540 (FL) and 0.289/0.349/0.414/0.517 (RL) (Fig. 7, Additional file 6: Fig. S5, 6). Comparing the difference in aSNN between late-stage low/middle activity and high/ultra-high activity, we see that for RL aSNN was higher for high/ultra-high, whereas for FL aSNN was higher for low/middle activity compounds.

Furthermore we compared aSNN results of FL and RL for in-house dataset in the same manners; all of 5,000 generated against middle stage compounds: 0.331/0.318/0.301/0.251 (FL) and 0.431/0.425/0.427/0.348 (RL), top 500 generated against middle stage compounds: 0.510/0.507/0.499/0.405 (FL) and 0.431/0.425/0.427/0.348 (RL), top 100 generated against middle stage compounds: 0.559/0.570/0.577/0.459 (FL) and 0.470/0.442/0.456/0.387 (RL); all of 5,000 generated against late stage compounds: 0.288/0.277/0.273/0.239 (FL) and 0.376/0.357/0.361/0.311 (RL), top 500 generated against late stage compounds: 0.527/0.534/0.543/0.442 (FL) and 0.455/0.429/0.441/0.370 (RL), top 100 generated against late stage compounds: 0.563/0.579/0.602/0.489 (FL) and 0.490/0.464/0.479/0.394 (RL) (Fig. 7, Additional file 6: Fig. S5, 6). We see that for in-house datasets, the aSNN to the high/ultra-high activity compounds were mostly similar or less than that to low/middle activity compounds for both FL and RL. In the worst scenario, RL strategy failed to improve over FL such as the case of aSNN in top 100 and 500 generated compounds against the late-stage compounds (Additional file 6: Fig. S6). Hence, we conclude that for public dataset RL strategy can work well; however, for in-house dataset it has no benefit over FL in this evaluation. However, as discussed in the previous section the classification model accuracy may affect RL strategy (notably late-stage high-activity compounds in in-house projects B, D and F result in low predicted activity scores, Additional file 6: Fig. S3), influencing whether the generative model can highly active compounds or not.

### The effect of diversity filter and inception on rediscovery and aSNN

We next investigated whether variations of the protocol, namely the use of a diversity filter and inception, were able to improve performance metrics. Rediscovery obtained when using those options is shown in Additional file 6: Table S6. Compared with the result of RL, we could not find any beneficial effect of a DF and inception when evaluating rediscovery. The aSNN results across model options in the middle stage are shown in Additional file 6: Fig. S5 and those for late stage are shown in Additional file 6: Fig. S6. We also could not find any effect of DF and inception which contribute to an increase in the similarity of generated compounds to high or ultra-high activity compounds when evaluating aSNN. However, especially for DF, this option surely contributed to avoidance of mode collapse when evaluating uniqueness and novelty (Additional file 6: Table S4, 5) [59]. This can be seen by the increased variance in the learning curve in Additional file 6: Fig. S7 where the RL-DF model must consistently identify new areas of chemical space that maximize the scoring function, compared with that of RF in Additional file 6: Fig. S4. Additionally, we investigated aSNN between the generated compounds from RL with/without DF and high/ultra-high activity compounds at the late stage for every 50 step. The result is shown in Additional file 6: Fig. S8 where it can be seen that RL-DF kept low aSNN even when aSNN of RL increased according to the steps. So, the effect of DF should be thought as fitting for purpose, such as scaffold hopping. Regarding inception, it has also shown before that it could contribute to the lead optimization process [26]; however, in the current study there is no beneficial effect observed.

### Predictive cluster analysis

According to the investigation we performed so far, the generated compounds for the public dataset had better rediscovery ratio and aSNN than the in-house dataset (Figs. 6, 7). Firstly, we investigated the real activity of each compound, and concluded that the public dataset which was transformed on a pseudo-time axis has an explicit relationship between activity and pseudo time; however, for the in-house dataset this relationship was much less profound (Fig. 5). In order to investigate this difference from the viewpoint of each compound’s topology and predictivity for late-stage active compounds, we further analysed the public and in-house dataset by counting the compound’s number in each region α, β, and γ after clustering by k-means. This analysis was meant to show whether each project had predictive or unpredictive clusters for the generative model. Specifically, if the number of compounds coming from α is not zero (i.e., active early stage compounds are contained in a given cluster) and the number of compounds from γ (i.e., late-stage active compounds) is higher than the number of compounds from β (i.e. late-stage inactive compounds), this is a ‘predictive’ cluster for the generative model, in the sense that the chemical space of the late-stage, desirable compounds is present in the early stage for the generative model (and vice versa for ‘unpredictive’ clusters). Table 1 shows the results of this analysis. It can be seen that for the public projects, most projects had more predictive clusters than unpredictive (the numbers of clusters which predictive/unpredictive in DRD2 was 7/1, for GSK this was: 4/4, for CDK2 4/3, for EGFR 5/4, and for ADRB2 5/5). On the other hand, in in-house projects, all of the models had more unpredictive clusters than predictive (the numbers of cluster predictive/unpredictive in project A was 0/6, in project B 1/5, in project C 1/6, in project D 0/5, in project E 0 /7, and in project F 1/3; Table 1). Even in the projects which have predictive clusters, the number of contents were highly biased; the compound’s number in each region α/β/γ for B of predictive cluster ID 6 were 112/0/2, those for C of predictive cluster ID 8 were 1/20/82, and those for F of predictive cluster ID 2 were 26/0/1. Based on this result, we conclude that the difference of rediscovery ratio and aSNN between the public and in-house projects might be based on whether projects have predictive clusters (and to which extent this is the case), or not. In this sense, we can formulate as a success criterion to utilize generative models in the drug discovery process is that we require seeds of promising compounds in the training dataset. Active learning might be a stepwise approach here. It should also be mentioned that the evaluation performed here is based on *known* actives only – hence, we were not able to evaluate the quality of potential ‘false positive’ compounds. We should note that this study focused on the ligand-based approach. Another method, that is free from QSAR modeling, is a structure-based approach, and recently there has been more research in this approach combined with generative models; see the references in detail [18, 39, 40, 60].

**Table 1:**
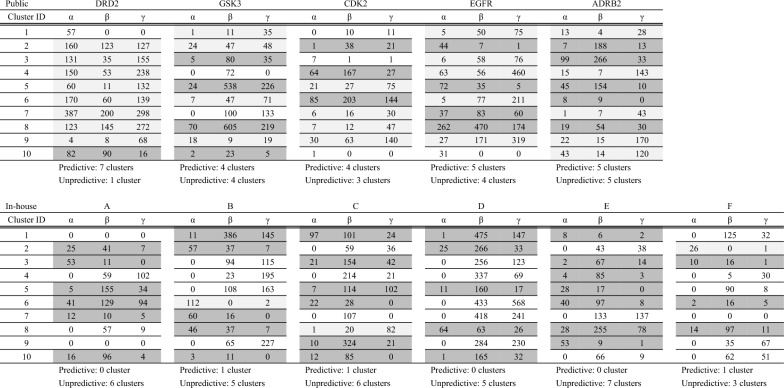
Clustering of real compounds (public and in-house dataset) by k-means (k = 10) and classification into α, β, and γ. If the number of α is not zero and that of γ is more than that of β, this seemed to be predictive of classification accuracy for the generative model (light grey column). On the other hand, if the number of α is not zero and that of γ is less than that of β, this seemed to be unpredictive (dark grey column)

### Analysis of negative log likelihood

Additionally, to investigate from another perspective of the difference of the result in the public and in-house dataset, we calculated NLL of real compounds located in α, β and γ with the prior network, the agent in FL, and the agent in RL. NLL reflects how likely the compound is to be sampled from the model (prior or agent); if the value is small, the compounds are more likely to be sampled from the model, and vice versa [26]. The result is shown in Additional file 6: Fig. S9, where it can be seen that in any dataset (public/in-house) and area (α, β and γ) the NLLs of the agent in FL were smaller than that of the agent in RL. This suggests that the FL was at least most likely to rediscover molecules from these subsets, however, in practice rediscovery rates were still very low. Focusing on the NLL of the agent in RL, in the public dataset (Additional file 6: Fig. S9a–e), the NLLs in γ were comparable to β; and in the in-house dataset (Additional file 6: Fig. S9f–k) the NLLs in γ slightly higher than that in β in project B, D, and F. This likely also reflects the observation of lower empirical classification scores for γ in these projects. Hence, we can conclude that compared with in-house dataset, in silico classification model of public dataset and therefore RL works better, and this reflects the better result of rediscovery and aSNN in public dataset than in in-house dataset.

### Case study of generated compounds’ structure (DRD2)

**Fig. 8:**
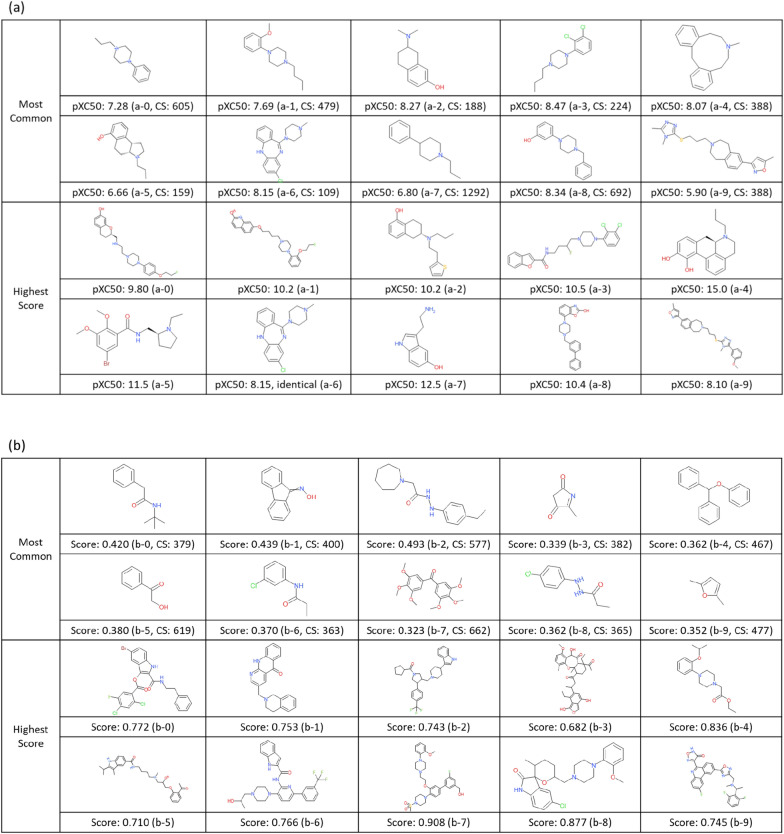

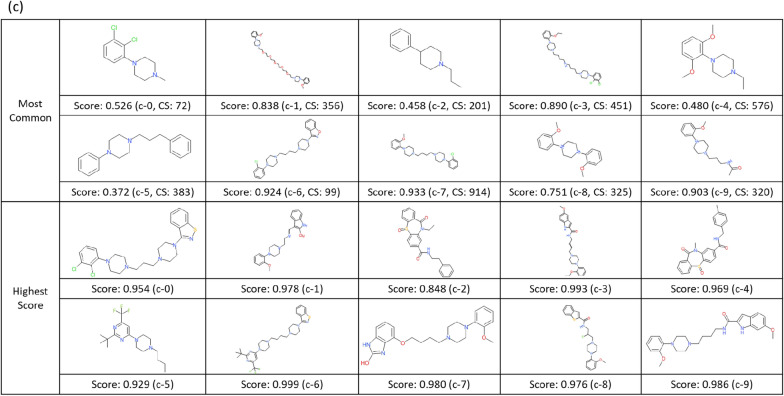
Example of DRD2 compounds. For the comparison of real (**a**) and generated compounds (**b**: from pre-trained prior model, **c**: from RL model) by visual inspection. The number after CS is the number of compounds included in the same cluster

In order to understand the chemistry of generated compounds better, we next examined it by visual inspections via clustering, using DRD2 ligands as an example. Representatives (most common structures (MCS) and highest scored structures (HSS)) from k-means clustering are depicted in Fig. 8. Compared to known ligands (Fig. 8a), there were some compounds that may require more careful consideration with regards to ‘drug like’ properties or synthetic ease. Regarding molecules from the pre-trained prior model (Fig. 8b) there were, (1) two small, fragment-like molecules present in the set (MCS b-3, MCS b-9), (2) many oxygen atoms present (MCS b-7, HSS b-3), (3) connections like hydrazine (MCS b-2 and b-8), and cycle to cycle connection by one attachment point like tetrahydropyran to pyrrolidine (HSS b-8). Regarding the generated compounds by RL (Fig. 8c) there were, (1) a few with long flexible chains (MCS c-1, MCS c-3), (2) sulfinyl groups present that are more commonly seen in antibiotics (HSS c-2, HSS c-4); however, there were no functional groups or idiosyncratic topologies without precedent in ChEMBL (i.e., the training data set). Therefore, the process of fine-tuning and RL using real chemical datasets had a beneficial effect on the generation of practical chemical structures.

## Conclusion

In this research we asked the question “*Can a generative model trained on early-stage project compounds generate middle/late-stage compounds *de novo?” To this end, we used experimental data from five public and six in-house project datasets as reference datasets and investigated the ability of different REINVENT protocols (i.e., focused learning, reinforcement learning, inception and diversity filter) to model the elapsed time of a synthetic expansion following hit identification. In all protocols, we found that late-stage compound rediscovery was very small for all datasets, however, rediscovery and aSNN to late-stage compounds was greater for public projects than that for in-house projects. Upon further investigation, the underlying aSNN between early- and middle/late-stage compounds in public projects was higher between active compounds than inactive compounds; however, for in-house projects the converse was true, thus posing a more conceptually difficult problem to model in-house datasets. We also investigated the effect of in silico classification model accuracy on reinforcement learning, identifying a weak positive correlation between classification accuracy and de novo aSNN to late-stage compounds; concerningly, late-stage compounds achieved low classification model scores highlighting a disparity in maximizing such a score in as in RL and in actually mimicking human-driven hit expansion. Despite these observations, RL outperformed FL on aSNN for public datasets. Predictive cluster analysis was performed further identifying that compounds from in-house datasets did have fewer predictive clusters than in public dataset, supporting the observation of RL having no benefit over FL for in-house datasets. Considering the difference in results between public and in-house dataset, objectively evaluating de novo compound design is hence, based on the current study, difficult retrospectively, because RL is severely confounded by classification accuracy and applicability domain. From the practical perspective, we can formulate as a success criterion to utilize generative models in the drug discovery process is that we require seeds of promising compounds in the training dataset; active learning might be a stepwise approach here. At the same time, we have shown that the generative model recovers very few middle/late-stage compounds from *real-world* drug discovery projects, highlighting the fundamental difference between human and automated design, as well as the difference between single-objective and multi-parameter optimization, with the latter being the norm in real-world drug discovery projects.

### Supplementary Information

**Additional file 1.** Supporting_Dataset_1_DRD2.xlsx.

**Additional file 2.** Supporting_Dataset_2_GSK3.xlsx.

**Additional file 3.** Supporting_Dataset_3_CDK2.xlsx.

**Additional file 4.** Supporting_Dataset_4_EGFR.xlsx.

**Additional file 5.** Supporting_Dataset_5_ADRB2.xlsx.

**Additional file 6: Table S1.** Numbers of datasets used for this study. **Table S2.** Metrics of generated compounds by the focused learned and reinforcement generation model. **Table S3.** Validity of each run. **Table S4.** Uniqueness of each run. **Table S5.** Novelty of each run. **Table S6.** Rediscovery ratio of all of the runs. **Table S7.** Average of single nearest neighbour similarity (aSNN) between generated and middle/late stage’s test compounds. **Figure S1.** In silico classification model performance measured as balanced accuracy across public and proprietary projects. **Figure S2.** Accuracy of the in silico classification model and rediscovery/aSNN. **Figure S3.** Score of in silico classification model using real compounds located in α, β, and γ. **Figure S4.** Learning Curve of Each Target in RL. **Figure S5.** Average of single nearest neighbour similarity (aSNN) between generated compounds and test compounds for all projects in the middle stage. **Figure S6.** aSNN between generated compounds and test compounds for all projects in the late stage. **Figure S7.** Learning Curve of Each Target in RL-DF. **Figure S8.** aSNN of generated compound in each step from RL with/without DF to the high/ultra-high active compounds. **Figure S9**. Negative log likelihood of real compounds located in α, β, and γ with the prior network, the agent in FL, and the agent in RL

## Data Availability

The data that support the findings of this study are available on request from the corresponding author. All software used in this study was freely available, REINVENT2.0, https://github.com/MolecularAI/Reinvent/tree/reinvent.v.2.0; MolSore, https://github.com/MorganCThomas/MolScore.

## Abbreviations

aSNN: Average of single nearest neighbor
NLP: Natural language process,
MPO: Multiple parameters optimization
RNNs: Recurrent neural network
FCNNs: Fully connected neural network
CNNs: Convolutional neural network
VAE: Variational auto encoder
GAN: Generative Adversarial Networks
LSTM: Long-short time memory
DRD2: Dopamine Receptor D2
GSK3: Glycogen synthase kinase 3
CDK2: Cyclin-dependent kinase 2
EGFR: Epidermal Growth Factor Receptor
ADRB2: Anrenergic receptor beta2
FL: Focused learned agent network
RL: Reinforcement learning
RF: Random forest
DF: Diversity filter
NLL: Negative log likelihood

## References

[1] Gómez-Bombarelli R (2018). Automatic chemical design using a data-driven continuous representation of molecules. ACS Cent Sci.

[2] Thomas M (2022). Applications of artificial intelligence in drug design: opportunities and challenges. Methods Mol Bio.

[3] Scannell JW, Bosley J (2016). When quality beats quantity: decision theory, drug discovery, and the reproducibility crisis. PLoS ONE.

[4] Plowright AT (2012). Hypothesis driven drug design: improving quality and effectiveness of the design-make-test-analyse cycle. Drug Discovery Today.

[5] Danziger DJ, Dean PM (1989). Automated site-directed drug design: a general algorithm for knowledge acquisition about hydrogen-bonding regions at protein surfaces. Proceed Royal Soc London Series B Bio Sci.

[6] Douguet D, Thoreau E, Grassy G (2000). A genetic algorithm for the automated generation of small organic molecules: drug design using an evolutionary algorithm. J Comput Aided Mol Des.

[7] Schneider G, Lee ML, Stahl M, Schneider P (2000). De novo design of molecular architectures by evolutionary assembly of drug-derived building blocks. J Comput Aided Mol Des.

[8] Pandey M (2022). The transformational role of GPU computing and deep learning in drug discovery. Nature Machine Intelligence.

[9] Gawehn E, Hiss JA, Brown JB, Schneider G (2018). Advancing drug discovery via GPU-based deep learning. Expert Opin Drug Discov.

[10] Vamathevan J (2019). Applications of machine learning in drug discovery and development. Nat Rev Drug Discovery.

[11] Vogt M (2023). Exploring chemical space—Generative models and their evaluation. Artifi Int Life Sci.

[12] Polykovskiy D (2020). Molecular sets (MOSES): a benchmarking platform for molecular generation models. Front Pharmacol.

[13] Preuer K, Renz P, Unterthiner T, Hochreiter S, Klambauer G (2018). Fréchet ChemNet distance: a metric for generative models for molecules in drug discovery. J Chem Inf Model.

[14] Bender A (2022). Evaluation guidelines for machine learning tools in the chemical sciences. Nat Rev Chem.

[15] https://cache-challenge.org/ (access date: December 2nd, 2022)

[16] Brown N, Fiscato M, Segler MHS, Vaucher AC (2019). GuacaMol: benchmarking models for de novo molecular design. J Chem Inf Model.

[17] Gaulton A (2017). The ChEMBL database in 2017. Nucleic Acids Res.

[18] Thomas M, O’Boyle NM, Bender A, De Graaf C (2022) Re-evaluating sample efficiency in de novo molecule generation. https://arxiv.org/abs/2212.01385. 10.1186/s13321-022-00646-z PMC9531503 36192789

[19] Sheridan RP (2013). Time-split cross-validation as a method for estimating the goodness of prospective prediction. J Chem Inf Model.

[20] Bender A, Cortes-Ciriano I (2021). Artificial intelligence in drug discovery: what is realistic, what are illusions? Part 2: a discussion of chemical and biological data. Drug Discovery Today.

[21] Beckers M, Fechner N, Stiefl N (2022). 25 years of small-molecule optimization at novartis: a retrospective analysis of chemical series evolution. J Chem Inf Model.

[22] Ståhl N, Falkman G, Karlsson A, Mathiason G, Boström J (2019). Deep reinforcement learning for multiparameter optimization in de novo drug design. J Chem Inf Model.

[23] He J (2021). Molecular optimization by capturing chemist’s intuition using deep neural networks. J Cheminformat.

[24] Delaney J (2009). Modelling iterative compound optimisation using a self-avoiding walk. Drug Discov Today.

[25] Olivecrona M, Blaschke T, Engkvist O, Chen H (2017). Molecular de-novo design through deep reinforcement learning. J Cheminformat.

[26] Blaschke T (2020). REINVENT 2.0: an ai tool for de novo drug design. J Chem Inf Model.

[27] Popova M, Isayev O, Tropsha A (2018). Deep reinforcement learning for de novo drug design. Sci Adv.

[28] Sewak M, Sahay SK, Rathore H (2020). An overview of deep learning architecture of deep neural networks and autoencoders. J Comput Theor Nanosci.

[29] Bouwmans T, Javed S, Sultana M, Jung SK (2019). Deep neural network concepts for background subtraction: a systematic review and comparative evaluation. Neural Networ.

[30] Kearnes S, McCloskey K, Berndl M, Pande V, Riley P (2016). Molecular graph convolutions: moving beyond fingerprints. J Comput Aided Mol Des.

[31] De Cao T, Kipf T (2018). MolGAN: an implicit generative model for small molecular graphs. arXiv.

[32] Hochreiter S, Schmidhuber J (1997). Long short-term memory. Neural Comput.

[33] Chung J, Gulcehre C, Cho K, Bengio Y (2014) Empirical evaluation of gated recurrent neural networks on sequence modeling. 1–9.

[34] Vaswani A (2017). Attention is all you need. arXiv.

[35] Ertl P, Lewis R, Martin E, Polyakov V (2017). In silico generation of novel, drug-like chemical matter using the LSTM neural network. arXiv.

[36] He J (2022). Transformer-based molecular optimization beyond matched molecular pairs. J Cheminformat.

[37] Guo J (2021). DockStream: a docking wrapper to enhance de novo molecular design. J Cheminformat.

[38] Marques G (2021). De Novo design of molecules with low hole reorganization energy based on a quarter-million molecule DFT screen. J Phys Chem A.

[39] Thomas M, Smith RT, O’Boyle NM, de Graaf C, Bender A (2021). Comparison of structure- and ligand-based scoring functions for deep generative models: a GPCR case study. J Cheminformat.

[40] Thomas M, O’Boyle NM, Bender A, de Graaf C (2022). Augmented Hill-Climb increases reinforcement learning efficiency for language-based de novo molecule generation. J Cheminformat.

[41] Blaschke T, Bajorath J (2022). Fine-tuning of a generative neural network for designing multi-target compounds. J Comput Aided Mol Des.

[42] Blaschke T, Engkvist O, Bajorath J, Chen H (2020). Memory-assisted reinforcement learning for diverse molecular de novo design. J Cheminformat.

[43] Yoshimori A, Kawasaki E, Kanai C, Tasaka T (2020). Strategies for design of molecular structures with a desired pharmacophore using deep reinforcement learning. Chem Pharm Bull.

[44] Sun J (2017). ExCAPE-DB: an integrated large scale dataset facilitating Big Data analysis in chemogenomics. J Cheminformat.

[45] Sayers EW (2021). Database resources of the National Center for Biotechnology Information. Nucleic Acids Res.

[46] Sander T, Freyss J, von Korff M, Rufener C (2015). DataWarrior: an open-source program for chemistry aware data visualization and analysis. J Chem Inf Model.

[47] Ertl P, Patiny L, Sander T, Rufener C, Zasso M (2015). Wikipedia chemical structure explorer: substructure and similarity searching of molecules from Wikipedia. J Cheminformat.

[48] RD-kit: https://www.rdkit.org/docs/index.html# Access 5 June 2023

[49] Sousa T, Correia J, Pereira V, Rocha M (2021). Generative deep learning for targeted compound design. J Chem Inf Model.

[50] Rogers D, Hahn M (2010). Extended-connectivity fingerprints. J Chem Inf Model.

[51] Breiman L (2001). Random forests. Mach Learn.

[52] Du Y, Fu T, Sun J, Liu S (2022). MolGenSurvey: a systematic survey in machine learning models for molecule design. arXiv.

[53] Bjerrum EJ, Margreitter C, Blaschke T, de Castro RL-R (2023). Faster and more diverse de novo molecular optimization with double-loop reinforcement learning using augmented SMILES. J Comput Aided Mol Des.

[54] Segler MHS, Kogej T, Tyrchan C, Waller MP (2018). Generating focused molecule libraries for drug discovery with recurrent neural networks. ACS Cent Sci.

[55] Atance SR, Diez JV, Engkvist O, Olsson S, Mercado R (2022). De Novo drug design using reinforcement learning with graph-based deep generative models. J Chem Inf Model.

[56] Jasial S, Hu Y, Vogt M, Bajorath J (2016). Activity-relevant similarity values for fingerprints and implications for similarity searching. F1000Research.

[57] Hert J (2004). Comparison of topological descriptors for similarity-based virtual screening using multiple bioactive reference structures. Org Biomol Chem.

[58] Esposito C, Landrum GA, Schneider N, Stiefl N, Riniker S (2021). GHOST: adjusting the decision threshold to handle imbalanced data in machine learning. J Chem Inf Model.

[59] Putin E (2018). Reinforced adversarial neural computer for de novo molecular design. J Chem Inf Model.

[60] Lamanna G (2023). GENERA: a combined genetic/deep-learning algorithm for multiobjective target-oriented de novo design. J Chem Inf Model.

