# Supplementary material for: On the difficulty of validating molecular generative models realistically: a case study on public and proprietary data
Source: J Cheminform. 2023 Nov 21;15:112. doi: 10.1186/s13321-023-00781-1 (PMC10664602; doi:10.1186/s13321-023-00781-1)
Supplement: Supplementary file 6 — Additional file 6: Table S1. Numbers of datasets used for this study. Table S2. Metrics of generated compounds by the focused learned and reinforcement generation model. Table S3. Validity of each run. Table S4. Uniqueness of each run. Table S5. Novelty of each run. Table S6. Rediscovery ratio of all of the runs. Table S7. Average of single nearest neighbour similarity (aSNN) between generated and middle/late stage’s test compounds. Figure S1. In silico classification model performance measured as balanced accuracy across public and proprietary projects. Figure S2. Accuracy of the in silico classification model and rediscovery/aSNN. Figure S3. Score of in silico classification model using real compounds located in α, β, and γ. Figure S4. Learning Curve of Each Target in RL. Figure S5. Average of single nearest neighbour similarity (aSNN) between generated compounds and test compounds for all projects in the middle stage. Figure S6. aSNN between generated compounds and test compounds for all projects in the late stage. Figure S7. Learning Curve of Each Target in RL-DF. Figure S8. aSNN of generated compound in each step from RL with/without DF to the high/ultra-high active compounds. Figure S9. Negative log likelihood of real compounds located in α, β, and γ with the prior network, the agent in FL, and the agent in RL [file 13321_2023_781_MOESM6_ESM.docx]

Supporting Information

On The Difficulty of Validating Molecular Generative Models Realistically: A Case Study on Public and Proprietary Data

Koichi Handa^1,2*^, Morgan C. Thomas^1^, Michiharu Kageyama^2^, Takeshi Iijima^2^, and Andreas Bender^1*^

^1^Centre for Molecular Informatics, Department of Chemistry, University of Cambridge, Lensfield Road, Cambridge, CB2 1EW, UK

^2^Toxicology & DMPK Research Department, Teijin Institute for Bio-medical Research, Teijin Pharma Limited, 4-3-2 Asahigaoka, Hino-shi, Tokyo 191-8512, Japan

Supplementary Information

Table S1 Numbers of datasets used for this study

Table S2 Metrics of generated compounds by the focused learned and reinforcement generation model.

Table S3 Validity of each run

Table S4 Uniqueness of each run

Table S5 Novelty of each run

Table S6 Rediscovery ratio of all of the runs

Table S7 Average of single nearest neighbour similarity (aSNN) between generated and middle/late stage’s test compounds

Figure S1 *In silico* classification model performance measured as balanced accuracy across public and proprietary projects.

Figure S2 Accuracy of the *in silico* classification model and rediscovery/aSNN

Figure S3 Score of *in silico* classification model using real compounds located in α, β, and γ

Figure S4 Learning Curve of Each Target in RL

Figure S5 Average of single nearest neighbour similarity (aSNN) between generated compounds and test compounds for all projects in the middle stage

Figure S6 aSNN between generated compounds and test compounds for all projects in the late stage

Figure S7 Learning Curve of Each Target in RL-DF

Figure S8 aSNN of generated compound in each step from RL with/without DF to the high/ultra-high active compounds

Figure S9 Negative log likelihood of real compounds located in α, β, and γ with the prior network, the agent in FL, and the agent in RL

Table S1 Numbers of datasets used for this study

The thresholds for activity classes in most projects are less than 6 for low, over 6 to less than 7 for middle, over 7 to less than 8 for high, over 8 for ultra-high. In the project E and F, to keep balance of the numbers of compounds in each class, the threshold for activity class are less than 7 for low, over 7 to less than 8 for middle, over 8 to less than 9 for high, over 9 for ultra-high. For the periods in public projects, from the beginning of the compounds by 50%, 50% to 75%, 75% to 100% are classified into early, middle, late stage, respectively. In in-house projects, to keep balance of the numbers of compounds in each stage, 500 or about 1000 compounds from the beginning are selected for the stage of early, then the same number of next compounds are selected as the stage of middle. Finally, remained compounds are classified into the stage of late.


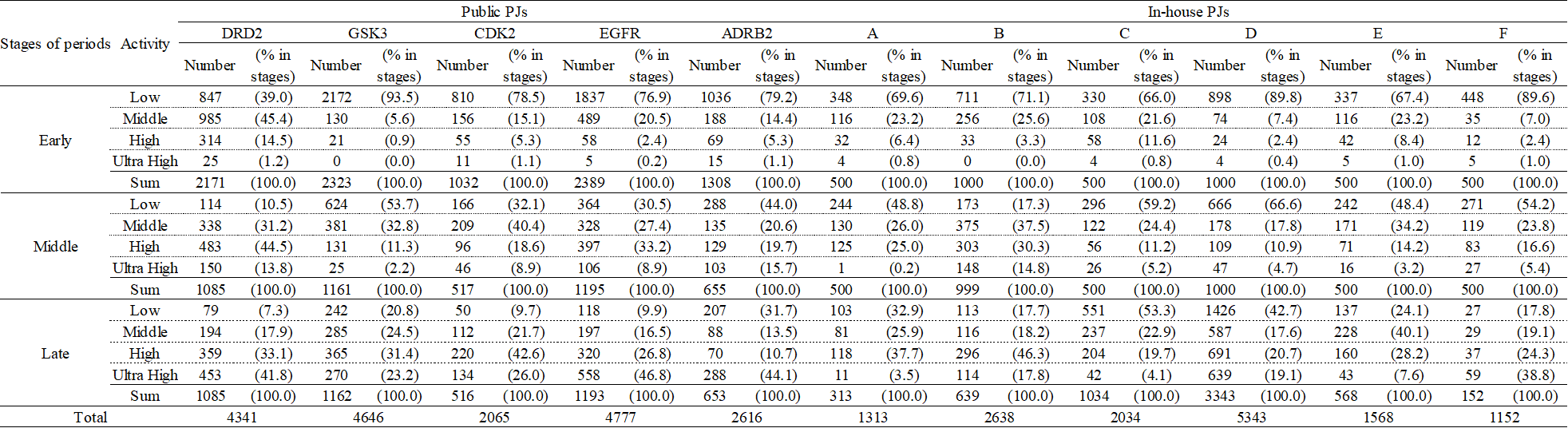


**Table S2 Metrics of generated compounds by the focused learned and reinforcement generation model.**

The validity of each target was over 98%. The uniqueness of public dataset was relatively high, from 39.4% (GSK3) to 82.2% (DRD2), and that of in-house dataset was low from 15.1% (F) to 50.9% (E). The novelty of each target was over 70%. The results of validity were almost the same of the result of Prior in the original article (97%); however, the novelty (90%) was lower than that. Although there was no description about uniqueness in the original article, that of the compounds in this study were not always high. Since those values of Control was over 90%, this suggested that the fine-tuning affects novelty and uniqueness largely.


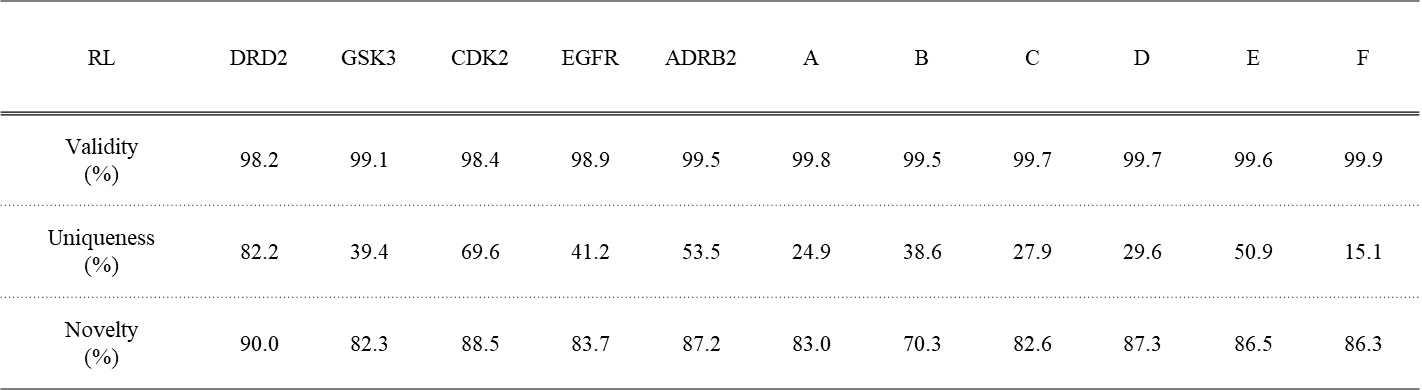


Table S3 Validity of each run

Through all of the runs, regardless of targets, the validities were high enough, over 95%.

Table S4 Uniqueness of each run

Through all of the projects, the uniqueness of the runs of RL-inception were lower than the other runs, from 36.4% (GSK3) to 59.8% (DRD2) in public dataset, and from 19.4% (F) to 40.8% (E) in in-house dataset. This problem was called mode collapse by RL. Then, inception was made it worse. However, if DF was used as an option, the low uniqueness was completely recovered [RL-DF: from 99.0% (B) to 99.8% (CDK2), RL-DF-inception: from 96.8% (B) to 98.5% (DRD2 and ADRB2)] (Table S 4). Consequently, it was considered that the options of DF could contribute to the recovery of uniqueness.


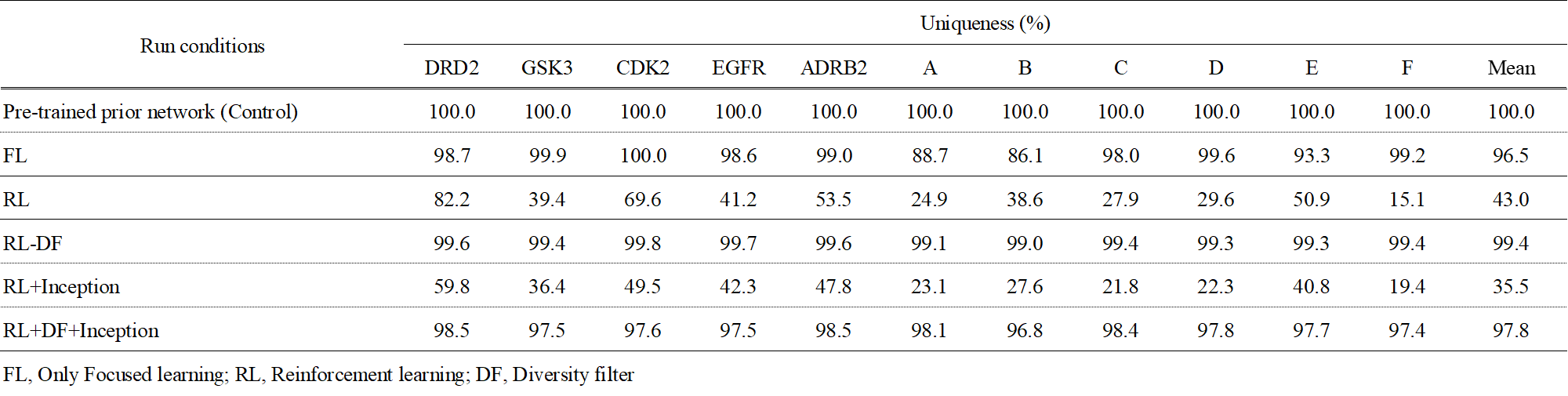


Table S5 Novelty of each run

Through all of the runs, regardless of targets, the novelties were high enough, over 70%.


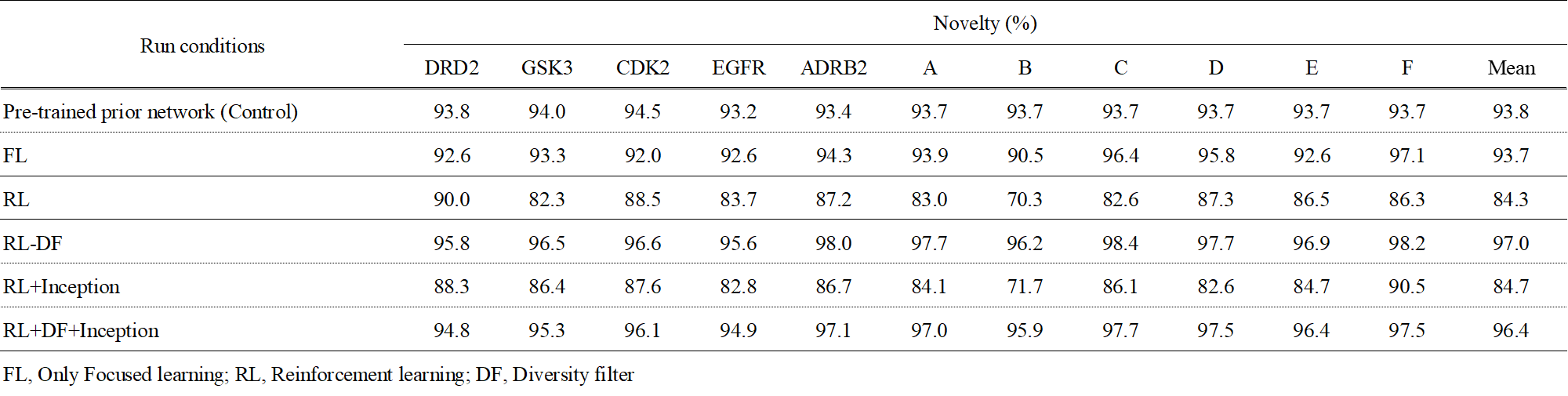


Table S6 Rediscovery ratio of all of the runs


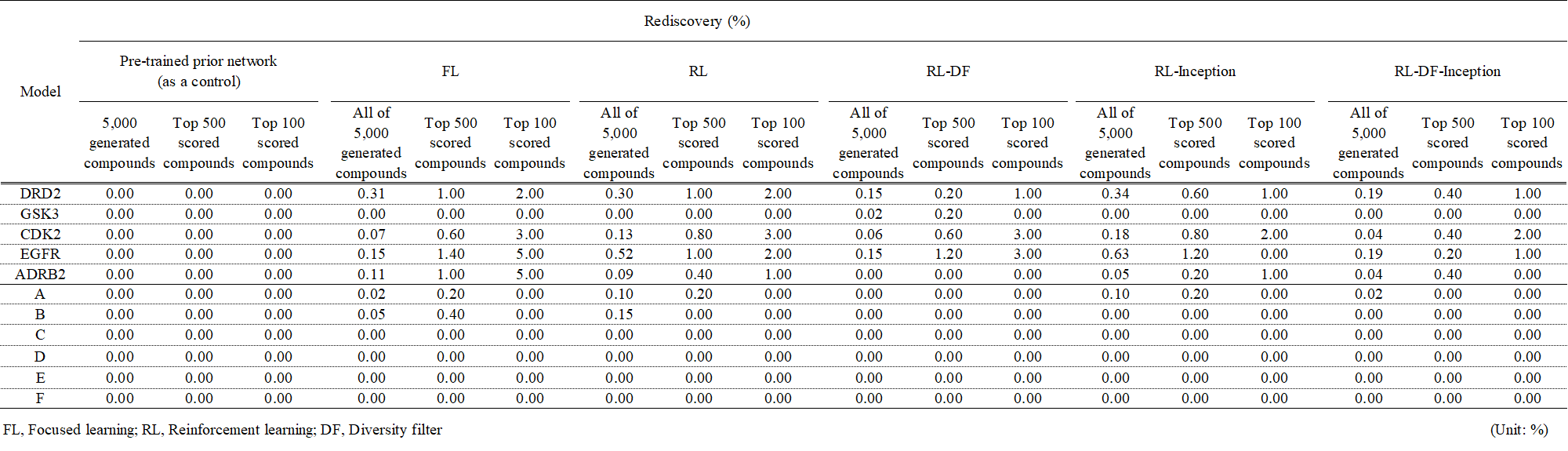


Table S7 Average of single nearest neighbour similarity (aSNN) between generated and middle/late stage’s test compounds

Supplementary Figures


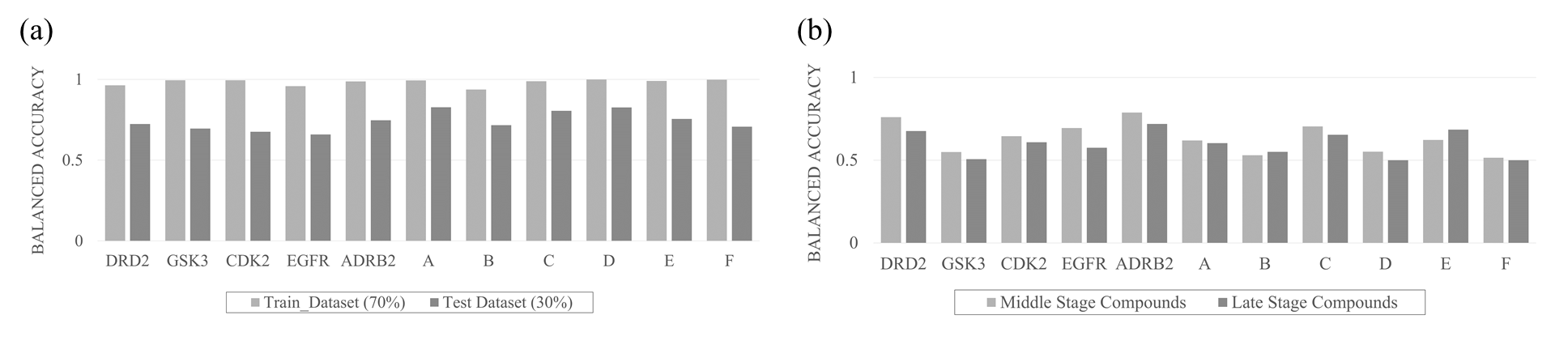


Figure S1 *In silico* classification model performance measured as balanced accuracy across public and proprietary projects.

It can be seen that *in silico* classification model performance has little impact on the performance of the resulting generative models. All of the compounds in the early stage are separated as training (70%) and test (30%) dataset in (a). Prospective analysis was performed using compounds in the middle and late stage in (b).


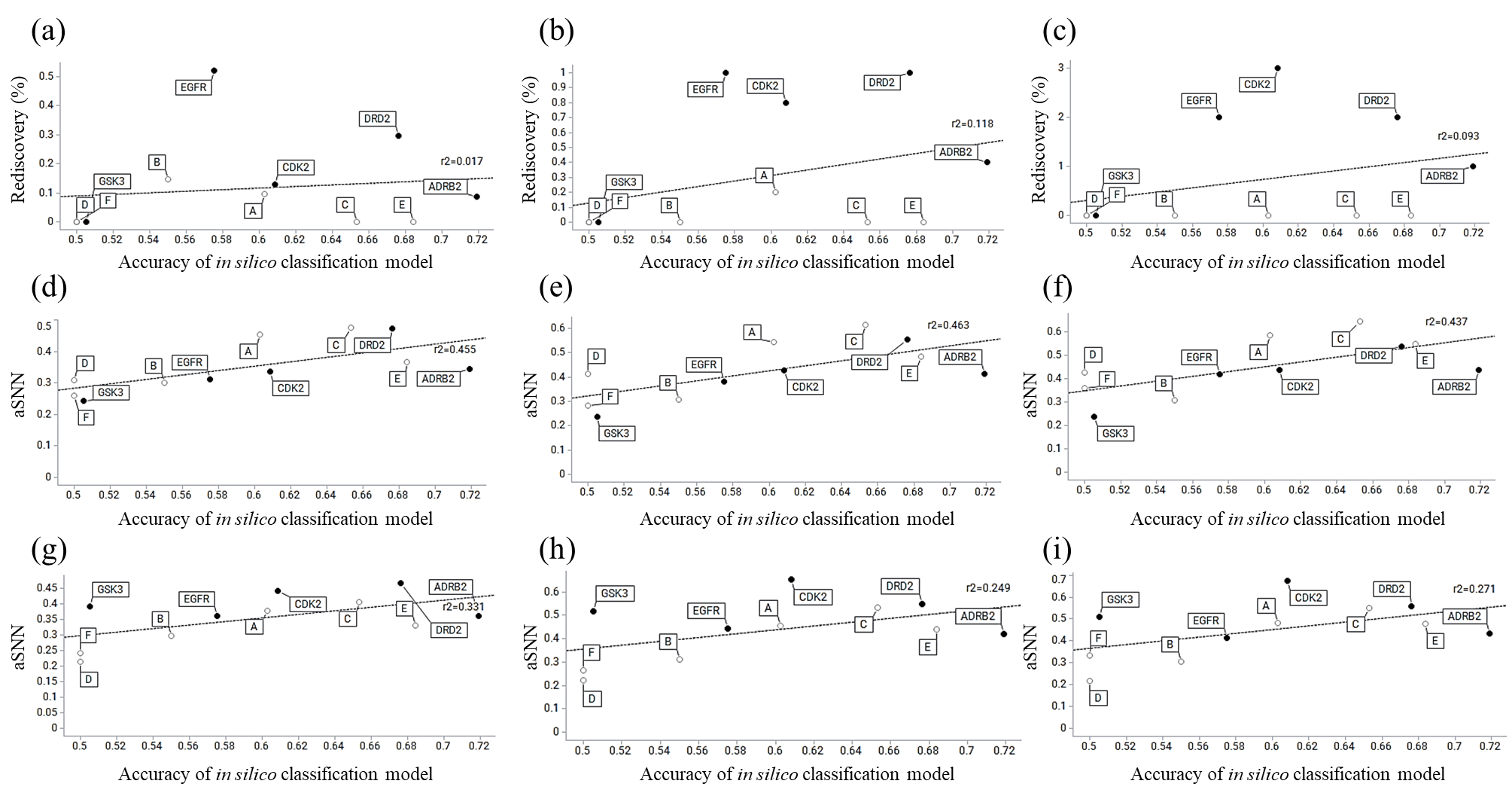


Figure S2 Accuracy of the *in silico* classification model and rediscovery/aSNN

The black and white circles represents public dataset (DRD2, GSK3, CDK2, EGFR and ADRB2) and in-house projects (A, B, C, D, E and F), respectively. Rediscovery (%) from (a) all of 5,000 generated compounds, (b) 500 top scored generated compounds, and (c) 100 top scored generated compounds. aSNN between generated compounds at the late stage and (d to f) high or (g to i) ultra-high activity real compounds. aSNN from (d, g) all of 5,000 generated compounds, (e, h) 500 top scored generated compounds, and (f, i) 100 top scored generated compounds.


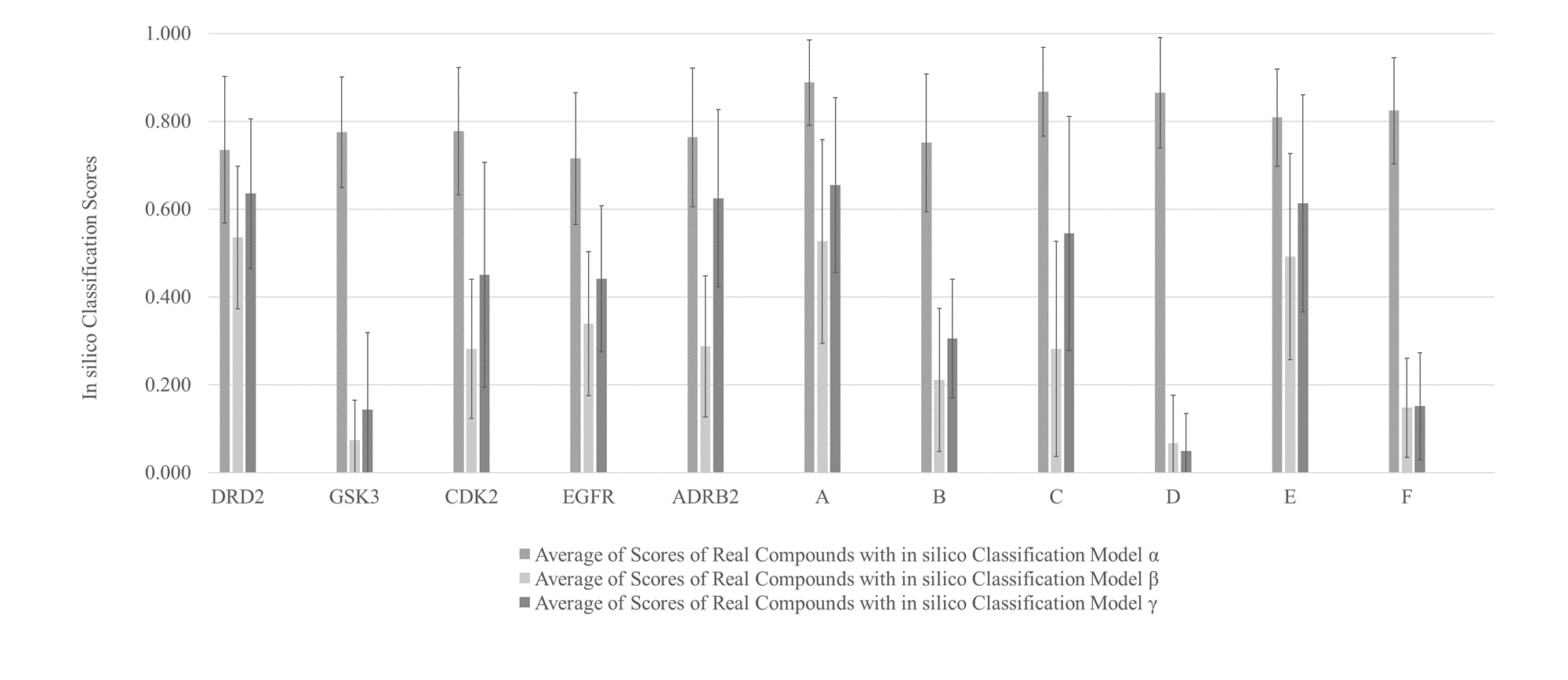


Figure S3 Score of *in silico* classification model using real compounds located in α, β, and γ

The compounds located in α were used as training dataset for the *in silico* model, consequently in all projects the scores were highest. Regarding β and γ, we can see the score of γ is clearly higher than that of β in most of projects other than D and F.


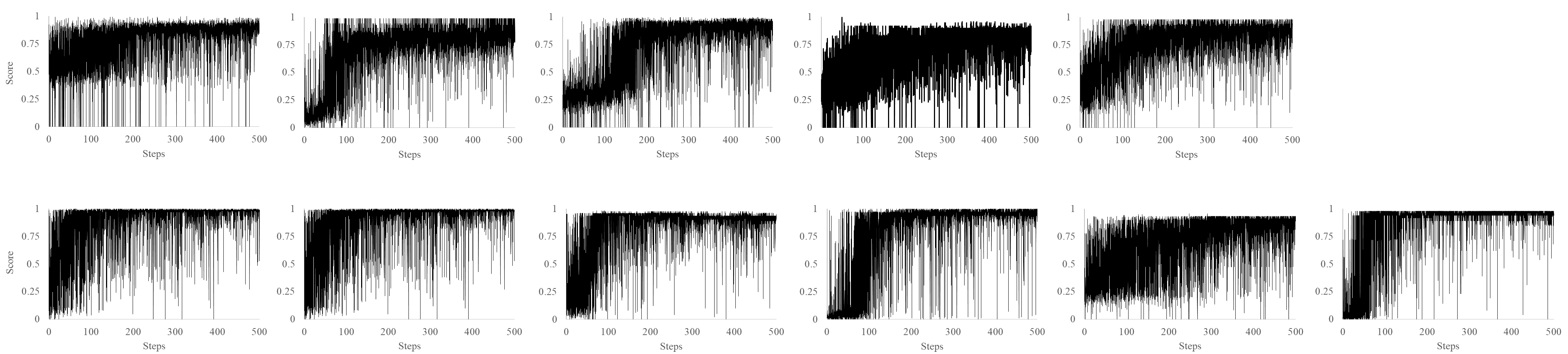


Figure S4 Learning Curve of Each Target in RL

These figures represent the target (in silico classification model) score (Y-axis) in RL model according to steps (X-axis). The upper row is for public dataset; (a) DRD2, (b) GSK3, (c) CDK2, (d) EGFR, (e) ADRB2. The lower row is for in-house dataset; (f) project A, (g) project B, (h) project C, (i) project D, (j) project E, (k) project F. All of the curves reached to high score close to 1.0.


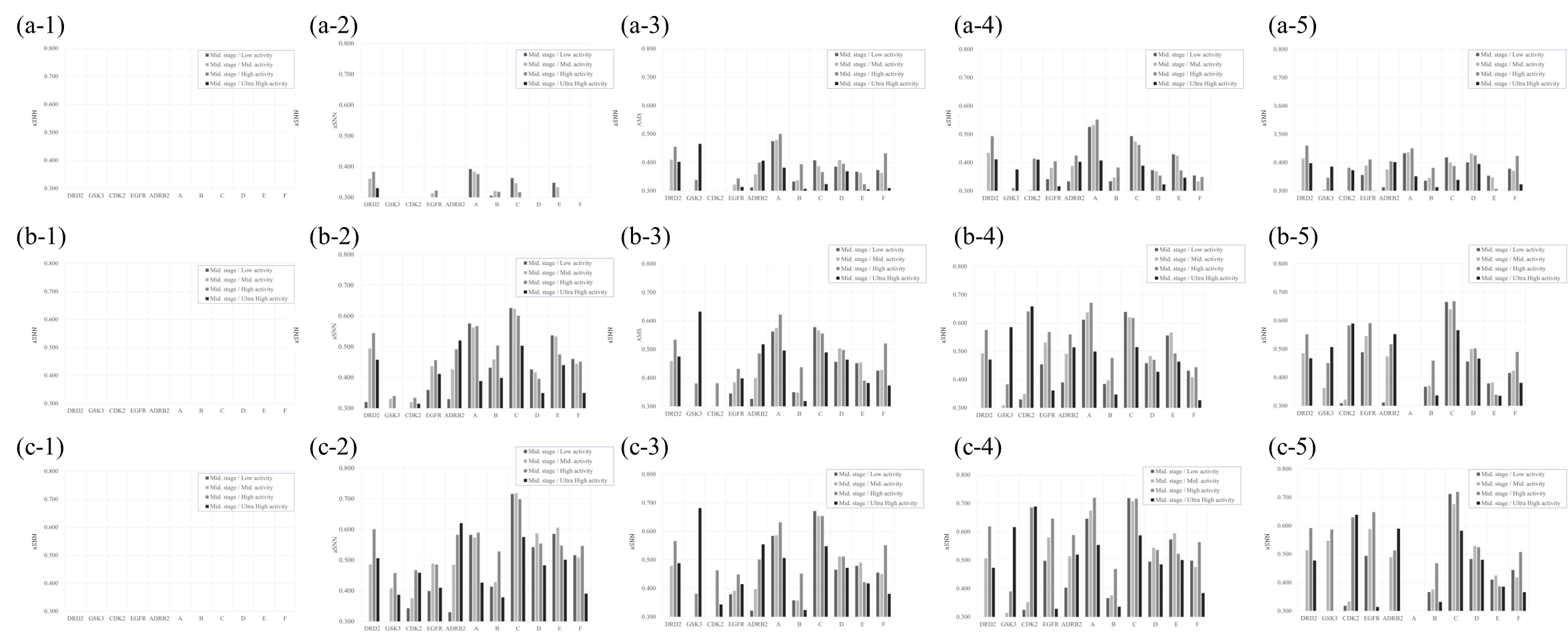


Figure S5 Average of single nearest neighbour similarity (aSNN) between generated compounds and test compounds for all projects in the middle stage

This figure indicates the aSNN in each run; (1) Pre-trained prior network (Control), (2) sampling from only focused learned agent network, (3) reinforcement learning (RL)-diversity filter (DF), (4) RL-Inception, (5) RL-DF-Inception. The aSNN between generated compounds and test compounds whose activity were more than high in middle. The generated compounds were selected as (a) all of 5,000 compounds, (b) top 500 scored compounds by in silico classification model, and (c) top 100 scored compounds by in silico classification model. Y axis indicates aSNN, and it starts from 0.3. Each bar represents aSNN of generated compounds from the model for low activity compounds (dense gray), middle activity ones (light grey), high activity ones (grey), and ultra-high activity ones (black).


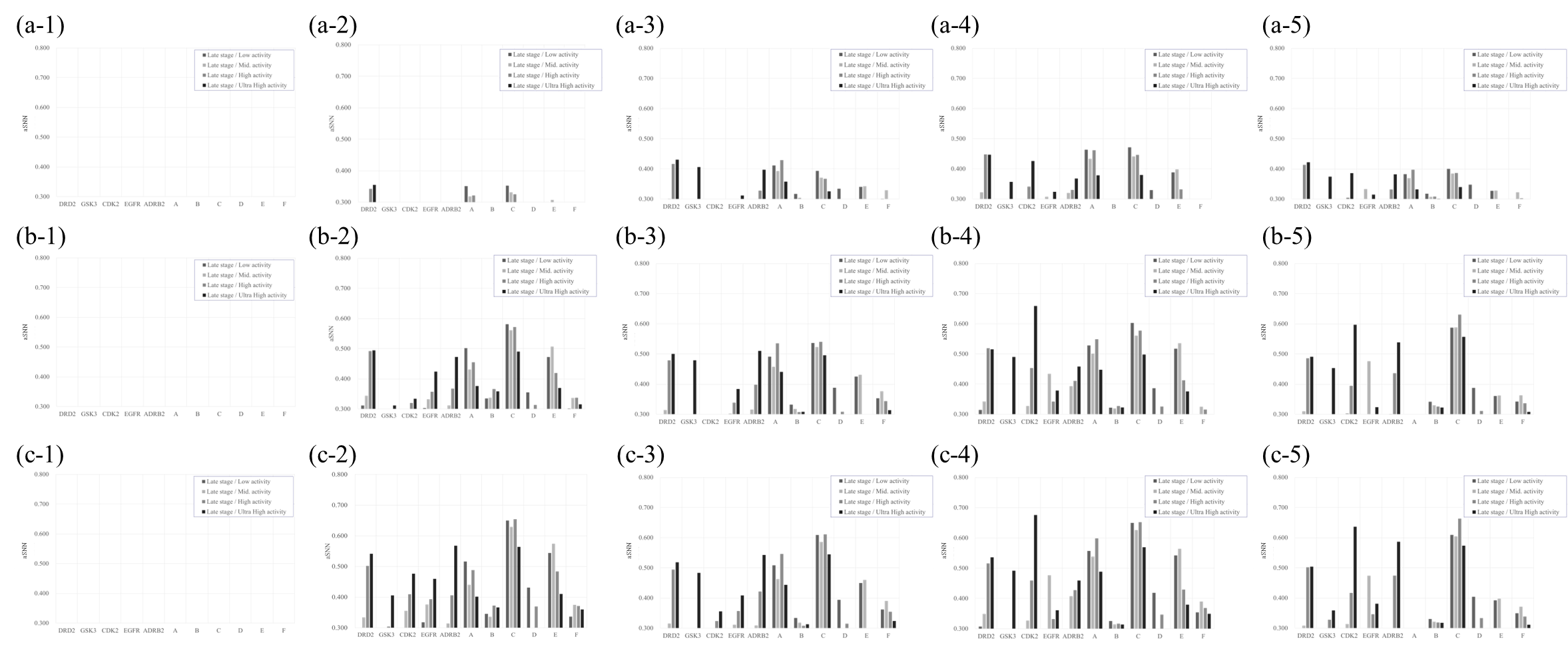


Figure S6 aSNN between generated compounds and test compounds for all projects in the late stage

This figure indicates aSNN in each run; (1) Pre-trained prior network (Control), (2) sampling from only focused learned agent network, (3) reinforcement learning (RL)-diversity filter (DF), (4) RL-Inception, (5) RL-DF-Inception. The aSNN between generated compounds and test compounds whose activity were more than high in middle. The generated compounds were selected as (a) all of 5,000 compounds, (b) top 500 scored compounds by in silico classification model, and (c) top 100 scored compounds by in silico classification model. Y axis indicates aSNN, and it starts from 0.3. Each bar represents aSNN of generated compounds from the model for low activity compounds (dense gray), middle activity ones (light grey), high activity ones (grey), and ultra-high activity ones (black).


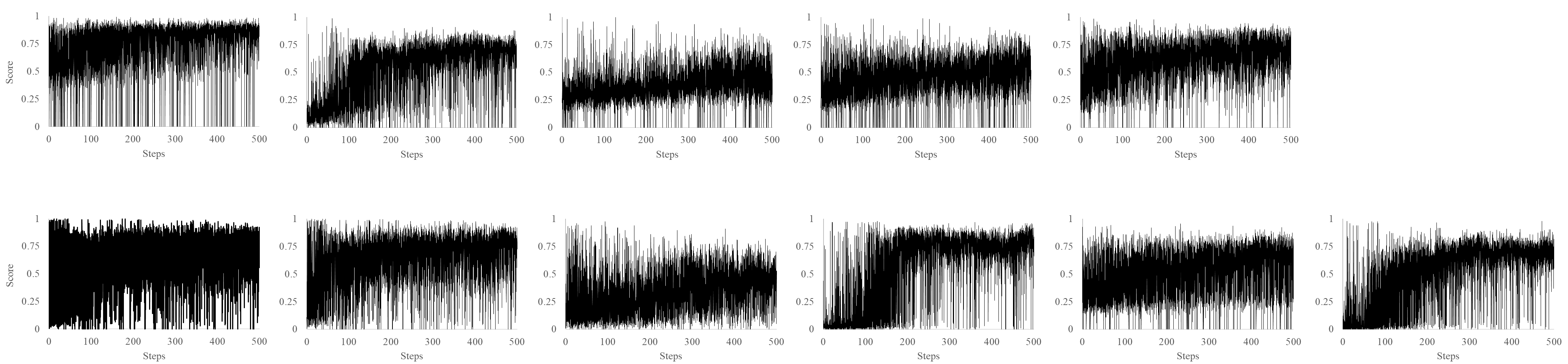


Figure S7 Learning Curve of Each Target in RL-DF

These figures represent the target (in silico classification model) score (Y-axis) in RL-DF model according to steps (X-axis). The upper row is for public dataset; (a) DRD2, (b) GSK3, (c) CDK2, (d) EGFR, (e) ADRB2. The lower row is for in-house dataset; (f) project A, (g) project B, (h) project C, (i) project D, (j) project E, (k) project F. Although all of the curves reached to high score, even in the latter steps, the scores fluctuated.


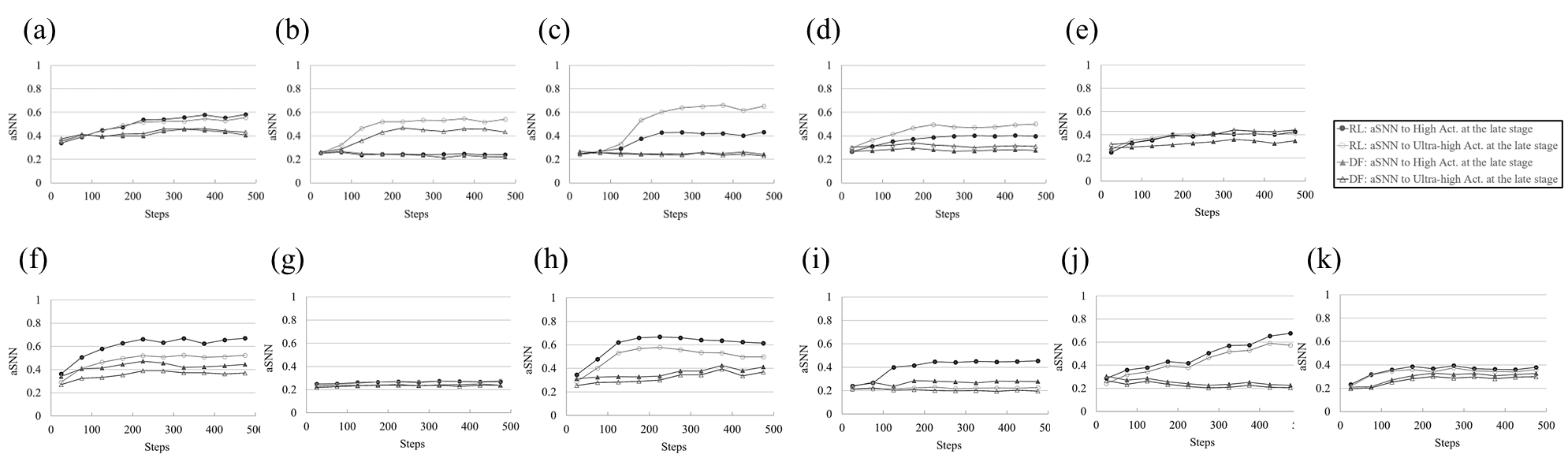


Figure S8 aSNN of generated compound in each step from RL with/without DF to the high/ultra-high active compounds

These figures represent the aSNN (Y-axis) according to every 50 step (X-axis). The upper row is for public dataset; (a) DRD2, (b) GSK3, (c) CDK2, (d) EGFR, (e) ADRB2. The lower row is for in-house dataset; (f) project A, (g) project B, (h) project C, (i) project D, (j) project E, (k) project F. The black circle represents aSNN between the generated compounds from RL and high activity real compounds at the late stage. The white circle represents aSNN between the generated compounds from RL and ultra-high activity real compounds at the late stage. The gray triangle represents aSNN between the generated compounds from RL-DF and high activity real compounds at the late stage. The white triangle represents aSNN between the generated compounds from RL and ultra-high activity real compounds at the late stage. In most cases aSNN from RL-DF remains lower than RL.


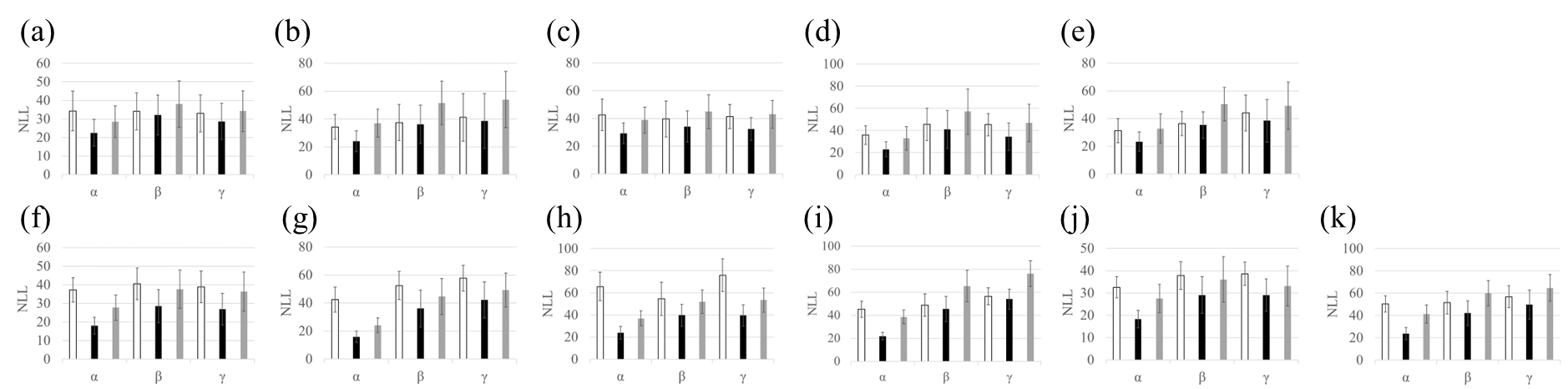


Figure S9 Negative log likelihood of real compounds located in α, β, and γ with the prior network, the agent in FL, and the agent in RL

The upper row is for public dataset; (a) DRD2, (b) GSK3, (c) CDK2, (d) EGFR, (e) ADRB2. The lower row is for in-house dataset; (f) project A, (g) project B, (h) project C, (i) project D, (j) project E, (k) project F. The negative log likelihood (NLL) with the prior network, the agent in FL, and the agent in RL are shown as white, black, and gray bars, respectively. In any project and cases (α, β, and γ), NLL of the agent in FL were smaller than the agent in RL.
